# HPTN 071 (PopART): A Cluster-Randomized Trial of the Population Impact of an HIV Combination Prevention Intervention Including Universal Testing and Treatment: Mathematical Model

**DOI:** 10.1371/journal.pone.0084511

**Published:** 2014-01-15

**Authors:** Anne Cori, Helen Ayles, Nulda Beyers, Ab Schaap, Sian Floyd, Kalpana Sabapathy, Jeffrey W. Eaton, Katharina Hauck, Peter Smith, Sam Griffith, Ayana Moore, Deborah Donnell, Sten H. Vermund, Sarah Fidler, Richard Hayes, Christophe Fraser, HPTN 071 (PopART) study team

**Affiliations:** 1 MRC Centre for Outbreak Analysis and Modelling, Department of Infectious Disease Epidemiology, Imperial College London, London, United Kingdom; 2 ZAMBART, University of Zambia, School of Medicine, Ridgeway Campus, Lusaka, Zambia; 3 Department of Clinical Research, London School of Hygiene and Tropical Medicine, London, United Kingdom; 4 Desmond Tutu TB Centre, Department of Paediatrics and Child Health, Stellenbosch University, Stellenbosch, South Africa; 5 Department of Infectious Disease Epidemiology, London School of Hygiene and Tropical Medicine, London, United Kingdom; 6 Business School, Imperial College London, South Kensington, London, United Kingdom; 7 FHI 360, Research Triangle Park, North Carolina, United States of America; 8 Vaccine and Infectious Disease Division, Fred Hutchinson Cancer Research Center, Seattle, Washington, United States of America; 9 Vanderbilt Institute for Global Health and Department of Pediatrics, Vanderbilt University, Nashville, Tennessee, United States of America; 10 Department of Medicine, Imperial College London, London, United Kingdom; National Institute of Allergy and Infectious Diseases, United States of America

## Abstract

**Background:**

The HPTN 052 trial confirmed that antiretroviral therapy (ART) can nearly eliminate HIV transmission from successfully treated HIV-infected individuals within couples. Here, we present the mathematical modeling used to inform the design and monitoring of a new trial aiming to test whether widespread provision of ART is feasible and can substantially reduce population-level HIV incidence.

**Methods and Findings:**

The HPTN 071 (PopART) trial is a three-arm cluster-randomized trial of 21 large population clusters in Zambia and South Africa, starting in 2013. A combination prevention package including home-based voluntary testing and counseling, and ART for HIV positive individuals, will be delivered in arms A and B, with ART offered universally in arm A and according to national guidelines in arm B. Arm C will be the control arm. The primary endpoint is the cumulative three-year HIV incidence.

We developed a mathematical model of heterosexual HIV transmission, informed by recent data on HIV-1 natural history. We focused on realistically modeling the intervention package. Parameters were calibrated to data previously collected in these communities and national surveillance data.

We predict that, if targets are reached, HIV incidence over three years will drop by >60% in arm A and >25% in arm B, relative to arm C. The considerable uncertainty in the predicted reduction in incidence justifies the need for a trial. The main drivers of this uncertainty are possible community-level behavioral changes associated with the intervention, uptake of testing and treatment, as well as ART retention and adherence.

**Conclusions:**

The HPTN 071 (PopART) trial intervention could reduce HIV population-level incidence by >60% over three years. This intervention could serve as a paradigm for national or supra-national implementation. Our analysis highlights the role mathematical modeling can play in trial development and monitoring, and more widely in evaluating the impact of treatment as prevention.

## Introduction

In 2011, the HPTN 052 trial (HPTN: HIV Prevention Trials Network) reported that early antiretroviral therapy (ART) reduces HIV-1 transmission amongst serodiscordant couples by 96% [1]. This finding, obtained in a closely monitored individually-randomized trial, corroborated the results of earlier studies [2], [3] and opened new and exciting perspectives for HIV prevention and control: expanding HIV testing and treatment could reduce sexual transmission of HIV close to zero [4]. A recent observational study in South Africa demonstrated that in fact, the ART coverage in the population immediately surrounding an individual was highly predictive of his/her risk of HIV acquisition [5]. In this context, several trials have been designed in order to test the feasibility of large scale HIV combination prevention strategies including universal HIV testing with immediate antiretroviral treatment for HIV-positive persons, and to measure their impact at the population level [6]–[10].

HPTN 071 (PopART, Population effects of Antiretroviral Therapy to reduce HIV transmission) is the largest of these trials, co-funded by the Office of the US Global AIDS Coordinator (OGAC), the US National Institutes of Health, and the Bill and Melinda Gates Foundation. It is planned to start in 2013, with annual follow-up until 2016, and analyses and results reported in 2017 [11]–[14].

In brief, it is a cluster-randomized trial consisting of 21 communities in Zambia and South Africa, covering approximately 1.2 million people. Each community, delimited as the catchment population of a health facility delivering ART, will be randomized to one of three arms. Interventions in arms A and B will include home-based voluntary testing (HBT) and counseling, male circumcision, prevention of mother to child transmission (PMTCT) services, treatment of sexually transmitted infections (STIs), condom distribution, and ART for HIV positive individuals. ART will be offered universally (regardless of CD4 count) in arm A and according to national guidelines (currently CD4<350 cell count per µL of peripheral blood) in arm B. Arm C will serve as a control arm with health system strengthening activities to ensure that standard of care services (voluntary testing and counseling, male circumcision, PMTCT, treatment of STIs, and ART for HIV positive individuals) are delivered according to national guidelines. The inclusion of three arms will allow separate assessment of the benefit of enhanced home-based voluntary testing, counseling and linkage to care, under national guidelines for treatment, and the additional prevention benefit of treatment regardless of CD4 count. The primary end-point will be cumulative HIV incidence over 3 years, measured in cohorts of 2,500 adults randomly selected in each of the 21 communities (total cohort size 52,500).

Mathematical modeling is an essential tool to assess the impact of interventions on HIV epidemics [15] because of the indirect benefit to members of the population not receiving the intervention. Also, mathematical modeling allows analyzing in a single framework the effect of multiple interventions, and thus takes into account synergistic (or interfering) effects between components of a combination prevention package. Therefore, over the last years, mathematical models have been increasingly used to provide insights in the potential long-term impacts of different interventions [4], [16], [17] and to assist with the post-hoc interpretation of trials and observational studies [18], [19]. It has also become clear that mathematical modeling could be used more extensively within clinical trials, to assist trial design, to inform monitoring and evaluation as a trial progresses, and finally to interpret and extrapolate the trial results [8].

Mathematical modeling was a key part of designing the HPTN 071 (PopART) trial: we developed a deterministic compartmental model of HIV transmission specifically conceived to assist the trial design. We focused on realistically describing the intervention package to be delivered during the trial. Model parameters were calibrated based on data collected during previous studies in the study communities as well as routine national surveillance data.

In the following, we describe this mathematical model and present the predicted impact of the intervention package that will be delivered during the trial. Most importantly, we present an extensive uncertainty and sensitivity analysis to quantify the influence of process variables (such as the uptake of testing) on the relative reduction in population level cumulative incidence in the intervention arms compared to the control arm. This analysis pinpoints the key variables that drive the magnitude of the reduction in incidence, and could therefore affect success or failure of this intervention package. Monitoring those variables during the trial will enhance evaluation of its progress, as will feeding values back into the model to obtain revised interim predictions.

## Materials and Methods

### Model structure

The model was designed with the intention to be simple but capable of representing different scenarios explored in trial design, and to represent a relative consensus of existing approaches to modelling the dynamics of generalised HIV epidemics. Its structure was particularly inspired by the models of Granich et al. [4], Hallett et al. [20], and Bezemer et al. [21], [22]. The model describes the generalised HIV epidemics in Zambia and South Africa, the two countries where the HPTN 071 (PopART) trial will take place.

The model is a deterministic compartmental model describing heterosexual transmission of HIV in the population aged 15 and over, specified by ordinary differential equations for the time-evolution of the number of individuals in different states. Our model is not age-structured and we therefore do not distinguish between the intervention, which is universal, and the measurement of incidence, which is in a cohort of adults aged 18 to 44. Our choice of age group was motivated by the availability of national prevalence estimates to which we calibrate our model.

A full description of the model structure, equations and parameterization, is presented in the supporting information (see in particular Figures S1 and S2 in File S1 and Tables S1, S2, S3 and S4 in File S1 for model structure and Tables S5, S6, S7, S8 and S9 in File S1 for definitions and values of model parameters).

**Figure 1:**
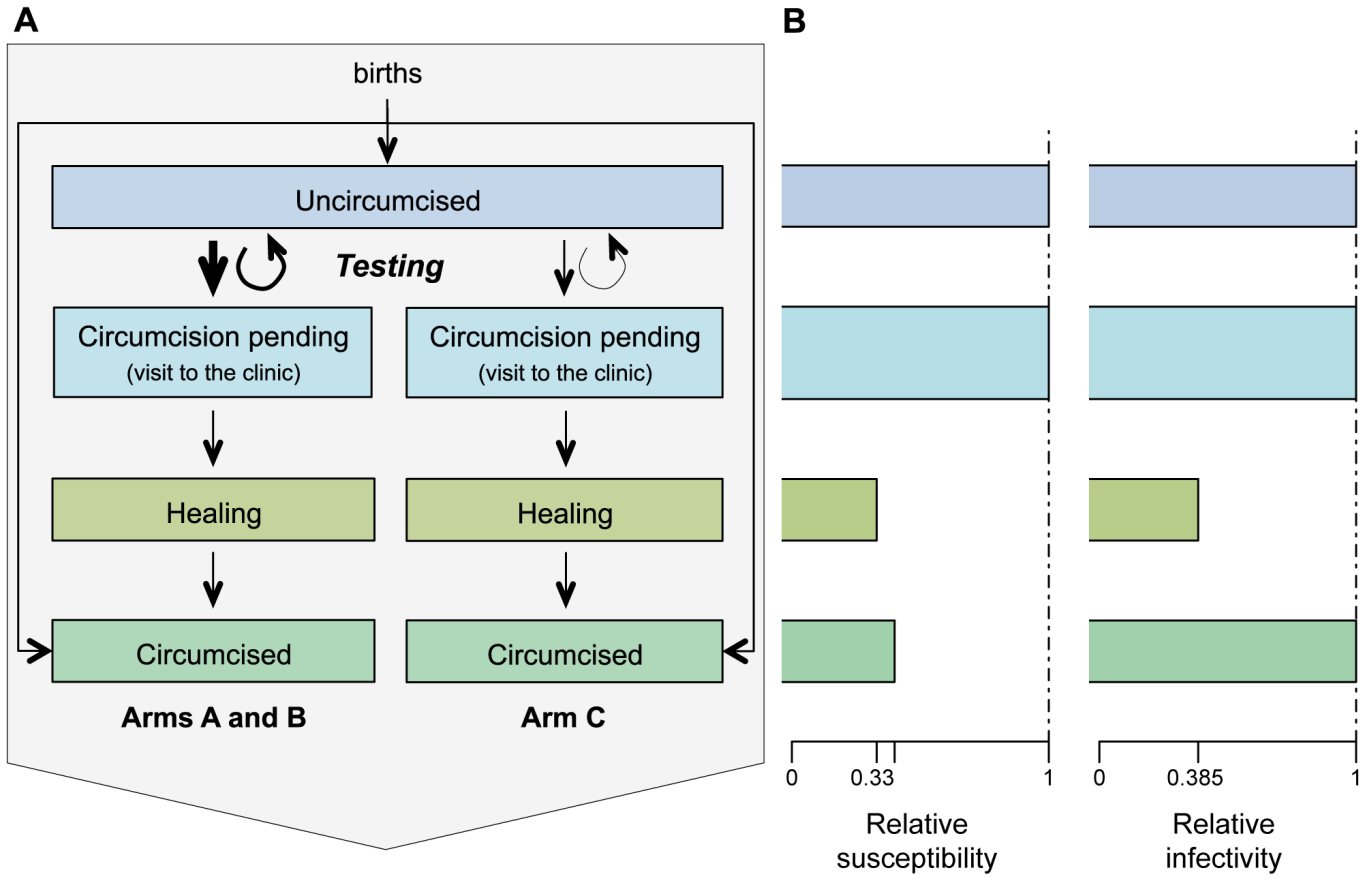
Model structure for susceptible men. **A.** Flow diagram of the model. Arrows depict the different flow rates between compartments. Men can be circumcised during childhood (*i.e.*, before the age of 15). If they are not, they can be circumcised following a negative HIV test. Upon testing, some HIV negative men decide to get circumcised. They then enter a “waiting” stage, which encompasses the time from testing to them actually visiting the clinic for circumcision. After circumcision, they go through a healing period, before being circumcised and healed. **B.** Relative susceptibility and infectivity in the different stages, relative to an uncircumcised man. Relative susceptibility and infectivity of circumcised men in healing period incorporate both biological increases in susceptibility and infectivity and reduction in sexual activity during the healing period (see main text).

**Figure 2:**
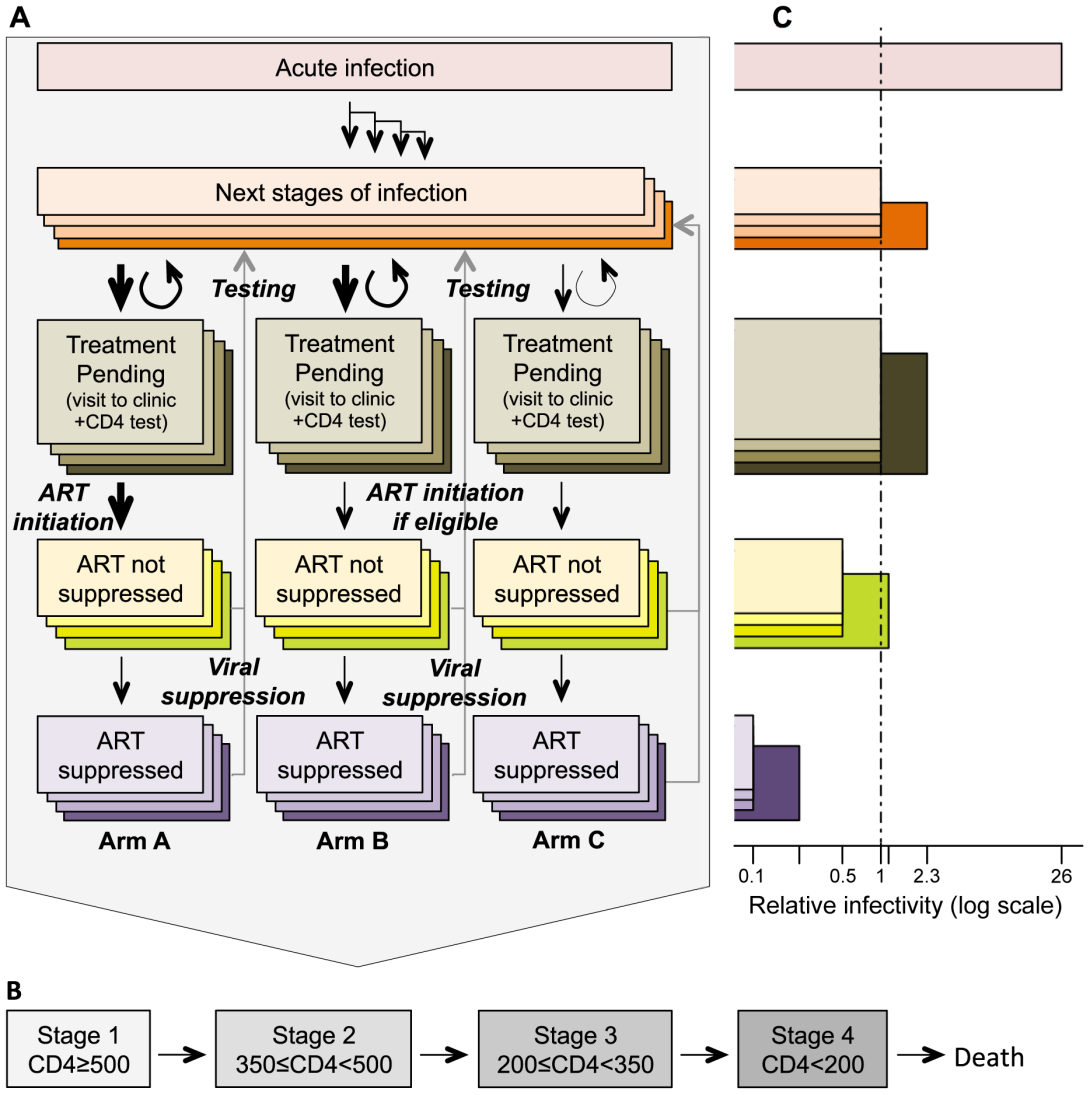
Model structure for infected individuals. **A.** Flow diagram of the model. The model describes progression through different stages of natural history and treatment. Arrows depict the different flow rates between compartments. Once infected, individuals enter an early/acute infection stage and then progress to death through 4 stages of infection shown in b. At any stage after acute infection, individuals can get HIV tested, following which a proportion of individuals will decide to seek care. Those individuals then enter the “treatment pending” stage, during which they visit the clinic and get assessed towards ART eligibility. Once assessed, all those eligible initiate treatment. Once on treatment, individuals go through a first phase during which viral load is not fully suppressed, before becoming successfully treated, that is, having a negligible viral load. Individuals on ART can drop out of or fail treatment; they then go back to the pre-test stages. **B.** Flow diagram of the four stages of infection following acute infection and leading to death. Those stages are defined by the level of CD4 in cells/mm3. Individuals can only move from a higher to a lower CD4 count. The rate of progressing through those stages is different for treated and untreated individuals (see File S1). **C.** Relative infectivity (on the log scale) of the different stages, compared to an undiagnosed individual with CD4≥350, not in acute infection. Individuals in acute infection and stage 4 (CD4<200) have an increased infectivity. Individuals on ART have a decreased infectivity.

Individuals are classified by sex (female/male), infection status (susceptible/infected), and sexual risk propensity (high/medium/low). The susceptible and infected stages are further stratified to represent the clinical progression of HIV and the intervention delivered in each arm of the trial. The model includes temporal delays between different steps of the intervention (such as testing and treatment). Susceptible males are classified as uncircumcised, uncircumcised planning circumcision (following a negative HIV test), circumcised in the wound healing period, and circumcised (see Figure 1A). Infected individuals are classified as untreated, untreated waiting for treatment (following a positive test), treated but not virally suppressed, and treated and virally suppressed. Infected individuals who are untreated are further classified in one of five disease stages: acute/early HIV, followed by four stages defined by the CD4 count (stage 1 corresponds to CD4≥500 cells/µL peripheral blood, stage 2 to 350≤CD4<500, stage 3 to 200≤CD4<350, and stage 4 to CD4<200, see Figure 1B). Upon ART initiation, infected individuals enter an ART category corresponding to the CD4 count level at which they initiated treatment, such that persons initiating treatment at higher CD4 levels have a better prognosis. A schematic description of the model for infected individuals is presented in Figure 2A.

### Modelling testing, treatment, and circumcision

We separately modelled a background level of HIV-related care for adults in all arms that would be presumed to occur in the absence of the trial activities, and an additional process, specific to interventions implemented in arms A and B during the trial.

### Background testing, treatment and circumcision

Background HIV testing was not modelled explicitly. Instead, we modelled the rate at which individuals initiate ART, encompassing both testing and successful linkage to care. We assume that only individuals with CD4<350 could initiate treatment. The rate at which they do so was modelled as a smooth function gradually ramping up from 2004 onwards, with a greater rate for individuals with CD4<200.

We assumed that, in all arms and both countries, a certain proportion of males are circumcised prior to entry into the modelled population at age 15. We assume these are fully circumcised. We did not model any adult circumcision outside of that offered as part of the intervention package in arms A and B.

### Additional testing, treatment and circumcision in arms A and B during the trial

During the trial, community HIV care providers teams (CHiPs) will offer, in arms A and B, home-based testing in annual rounds in all intervention communities. These intervention rounds are scheduled to last 6 months: here we model these taking place from 1^st^ July to 31^st^ December, from 2013 to 2015. Both the schedule and start date are subject to adjustment, with a likely 4 to 5-month delay from this modelled schedule. These annual rounds of testing were modelled by a constant number of tests offered each day by CHiPs. Following testing by CHiPs (which, when offered, is only accepted by a proportion of individuals), a fraction of men testing negative will decide to get circumcised and a fraction of individuals testing positive will decide to link to care (and start ART if eligible). Those individuals will go through the “waiting” stages (awaiting circumcision or awaiting treatment) before becoming circumcised or initiating treatment. Those stages were modelled to account for delays from the time that elapses between HIV testing and presentation at the health facility for circumcision or treatment initiation.

### Treatment failure and drop-out

Individuals on ART were assumed to stop receiving treatment (e.g. due to dropping out or treatment failure) at a rate of 10% per year (an assumption varied in sensitivity analysis). They were then assumed to go back to the “untreated” stage. They may then be re-started on treatment at a later time at the same rate as treatment-naïve persons.

### Clinical progression on and off treatment

Upon becoming infected, all persons first enter a period of acute HIV infection lasting for a mean of 2.9 months, after which infected persons may enter any of the CD4 cell count categories. The proportion entering each category and the rate of progression to the next lower CD4 count category were calibrated to recent clinical cohort data from the large multinational CASCADE collaboration [23] (see File S1). Compared to previous models, which assumed all individuals start with a post-seroconversion CD4 count ≥500 and progress through each CD4 stage, the new model better captures heterogeneity between individuals observed in the clinical seroconverter data.

Upon treatment initiation, following the approach of Granich et al. [4], individuals enter a ‘treatment’ compartment mirroring the CD4 stage from which they initiate treatment. They then progress through stages of treatment half as fast as untreated patients. This simple model allows capturing the improved prognosis for patients initiating treatment at higher CD4 cell counts [24]–[28]. Sensitivity analyses show that the short-term predicted epidemiological impact is not strongly dependent on assumptions about the rate of progression of individuals on treatment (see supplementary material in File S1). However, a more mechanistic representation of viral suppression and CD4 reconstitution [29] could be important for capturing long-term predictions of epidemiological impacts, costs, and clinical benefits. Interpretation of current clinical data from generalised epidemics in sub-Saharan Africa has proven difficult because of the confounding of mortality and loss to follow up [30]; improving these estimates will be an important feature of the analysis of trial outcomes in HPTN 071 (PopART), albeit with a relatively short time horizon.

### Contact patterns, relative susceptibility and relative infectivity

We use a model of assortative heterosexual sexual mixing between three sexual risk groups. Individuals in our model form partnerships at different rates according to their risk group. We assume that individuals in the low and middle risk groups have on average one new partner every ten years and one partner every year, respectively. The average number of partners per year for individuals in the high risk group is calibrated to fit national HIV prevalence estimates. We assume partnerships are made preferentially within the same risk group, with a level of assortativity which is calibrated by fitting the model to national HIV prevalence estimates. We also assumed that 5% of partnerships are formed with partners from outside the study community, thereby allowing for “contamination” of the intervention communities. We further assume that within a partnership, unprotected sexual acts occur at an instantaneous rate which depends on the risk groups of the two individuals: it is the same for all partnerships between individuals of different risk groups, as well as partnerships between two mid-risk individuals; it is twice as high for partnerships between two high-risk individuals and twice as low for partnerships between two low-risk individuals.

During the trial, we aim to collect data for better parameterisation of this component of the model.

We assumed that circumcision decreases male susceptibility by 60% [31]–[34]. We assume infectiousness is greater during acute/early and late stage infection, and reduced for individuals on ART (see Figure 1B, Figure 2C and Table S7 in File S1). Men in the wound healing period following circumcision are assumed to have decreased sexual activity, but an increased susceptibility and infectiousness per sex act, in balance leading to an overall reduced susceptibility and infectiousness during the same period than had they not undergone circumcision [35], [36]. We assume no difference in infectiousness for circumcised and healed infected males and uncircumcised infected males.

In a sensitivity analysis, we also investigate potential consequences of reductions in unprotected sexual activity (modelled as lower susceptibility and infectivity levels) for individuals in the “waiting” stages due to the HIV counselling and condom distribution.

### Model calibration

The non HIV-related death rate was calculated dynamically to constrain the population size and the birth rate to match national demographics data since 1978 (see File S1).

The HIV epidemic was calibrated to match HIV prevalence estimates reported by UNAIDS [37] by varying the basic transmission rate (*λ_0_*, the rate at which an untreated infected individual with CD4≥350 not in acute infection transmits to a partner, assuming they are both in the mid-risk group), the time of seeding of the epidemic, the proportion of individuals in each risk group, the rate of sexual contacts in the high risk group, and the assortativity. The background rate of ART initiation was modelled as a Hill function increasing from 2004 onwards to achieve the ART coverage data reported during the ZAMSTAR trial [38], [39].

### Uncertainty and sensitivity analysis

Uncertainty and sensitivity analyses were conducted to assess whether the predicted reduction in HIV incidence in the intervention arms was strongly influenced by the parameter values chosen to best fit the UNAIDS national prevalence estimates, and to analyse the impact that the “process” parameters, such as the uptake of circumcision during the intervention, would have on the estimated reduction in HIV incidence.

### Influence of parameters calibrated to prevalence curves

For each country, we used a Latin hypercube sampling scheme [40] to simulate epidemics for range of values for the parameters described in Table 1, and selected the 9 parameter sets (out of 9000) with best fits to the prevalence. For each of these, we then ran an optimization routine, starting from this parameter set, to obtain a neighbour parameter set with an improved fit to HIV prevalence. Because we were using a local optimisation algorithm, this did not converge on the global optimum. We compared the predicted reduction in incidence under these 9 final parameter sets to the original best-fit parameter combination.

**Table 1:** Sensitivity analysis: range of parameter values considered and influence on the relative reduction in 3-year cumulative incidence in arms A and B compared to arm C in each country.

Parameter	Name	Most pessimistic	Central target	Optimistic target	Most optimistic	Parameters of the linear regression*							
						ArmA				Arm B			
						Zambia		South Africa		Zambia		South Africa	
		(The range of values defined by the most pessimistic and most optimistic targets was uniformly explored)				Slope	%var**	Slope	%var**	Slope	%var**	Slope	%var**
Intercept	-	-	-	-	-	0.42	-	0.47	-	0.54	-	0.62	-
Relative infectivity due to behavioural changes at the community level	*i_bc_*	1.33	1.0	1.0	0.67	−0.32	33%	−0.32	34%	−0.54	81%	−0.58	86%
Relative infectivity of individuals under ART during the intervention, principally determined by adherence to ART	*i_ART_*	0.2	0.1	0.05	0.01	−1.1	34%	−1.1	35%	−0.60	8.4%	−0.68	8.9%
Probability of accepting HIV test when offered by CHiPs	*p_test_*	0.6	0.837	0.867	0.95	0.43	17%	0.41	16%	0.22	3.7%	0.20	2.7%
Probability of going to get treatment given a positive HIV test delivered by CHiPs	*p_ART_*	0.7	0.837	0.867	0.95	0.35	6.2%	0.35	6.5%	0.10	0.57%	0.15	0.89%
Proportion of sex acts with individuals from neighbouring areas	*π*	0.1	0.05	0.05	0.0	−0.71	3.7%	−0.70	3.6%	−0.19	0.21%	−0.27	0.27%
Annual drop-out rate after intervention has started	*φ_UTT_*	0.2	0.1	0.1	0.01	−0.26	1.7%	−0.29	1.9%	−0.11	0.27%	−0.15	0.24%
Probability of getting circumcised given negative HIV test delivered by CHiPs	*p_circ_*	0.25	0.5	0.5	0.75	0.096	1.5%	0.028	0.27%	0.17	4.4%	0.057	0.39%
Mean time between positive test and actual start of treatment (year)	*υ_wait_*	2/13 (8 weeks)	1/13 (4 weeks)	3/52 (3 weeks)	8/365 (8 days)	−0.016	0.098%	−0.028	0.035%	−0.014	0.16%	−0.027	0.054%
Relative susceptibility of individuals waiting circumcision	*s_pcirc_*	1	1	1	0.8	−0.0050	0.040%	−0.0050	0.013%	−0.0075	0.005%	0.0027	0.0095%
Average Rate of getting circumcised once decision to get circumcised has been made (year^−1^)	*τ_circ_*	6 (average delay from test to circumcision 2 months)	26 (average delay 2 weeks)	52 (average delay 1 week)	182.5 (average delay 2 days)	−0.000011	0.033%	0.00000016	0.084%	0.000022	0.040%	0.00000069	0.022%
Relative infectivity of individuals waiting treatment	*i_pART_*	1	1	1	0.8	−0.017	0.014%	−0.0037	0.059%	0.0095	0.025%	−0.0011	0.060%

Regression exploring the linear dependence of the relative reduction in 3-year cumulative incidence in arms A and B compared to arm C (on the linear-scale) on process parameters (see File S1 for detail).

%var: proportion of variance in the reduction in 3-year cumulative incidence explained by each predictor (see File S1 for detail).

### Influence of process parameters

To explore the influence of process parameters that could potentially be controlled during the intervention implementation, we defined four scenarios ranging from best to worst case (most optimistic, optimistic, central and most pessimistic), with corresponding parameters shown in Table 1. For each country, we generated, using a Latin hypercube sampling scheme [40], a set of 1000 parameters drawn uniformly within the range defined by the worst and best cases, and examined the resulting variability in the predicted 3-year cumulative HIV incidence in each arm. In order to assess the main drivers of this variability, we used a linear model exploring the relationship between the reduction in 3-year cumulative HIV incidence in intervention arms and the process parameters. The relative impact of each process parameter on the reduction in incidence was assessed by examining the proportion of the variance explained by each predictor (see File S1).

## Results

**Figure 3:**
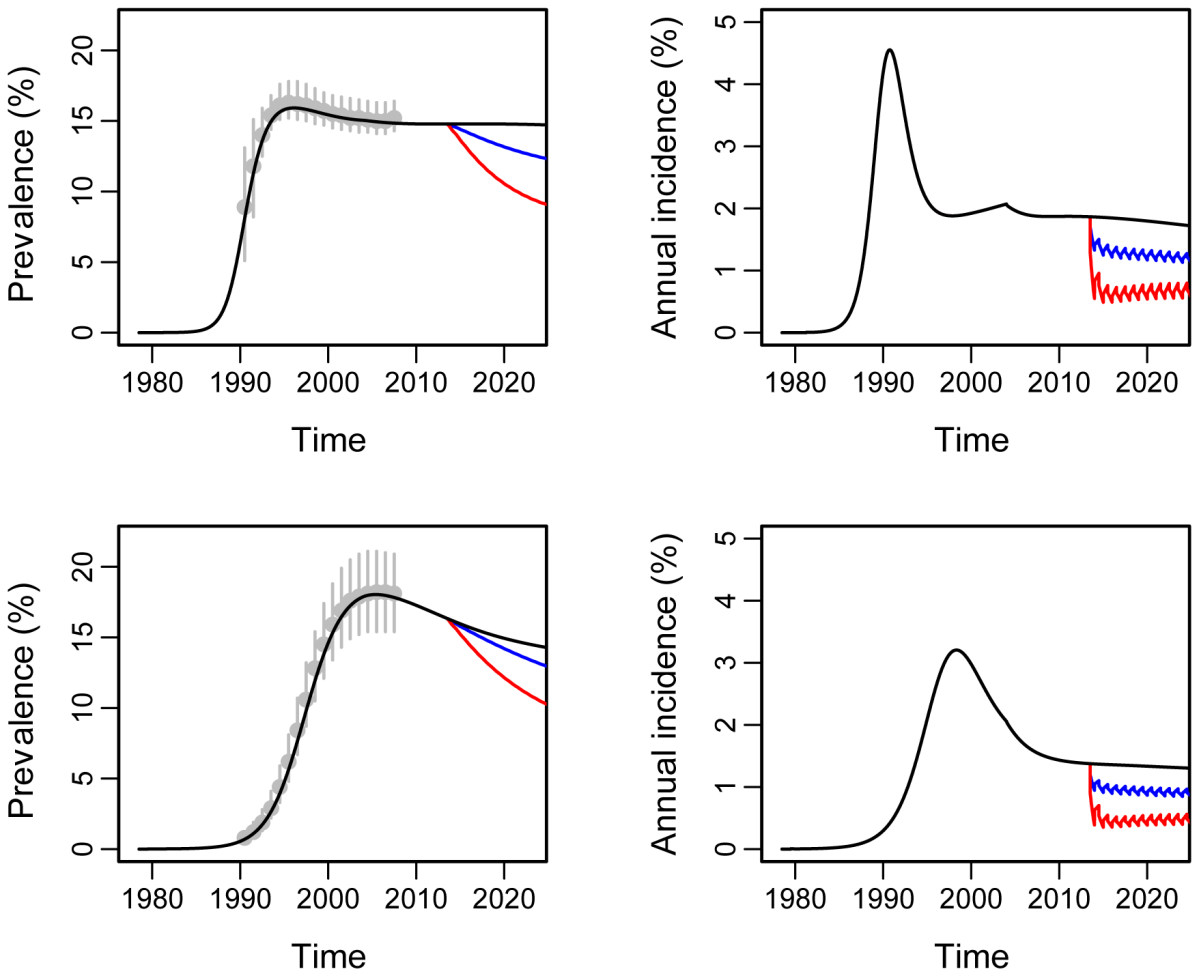
Model fit and projections under central target scenario for Zambia (top row) and South Africa (bottom row). Left panels show HIV prevalence and right panels show annualized HIV incidence over time. The red, blue and black lines correspond to arms A, B and C respectively. The grey dots and error bars are the UNAIDS HIV prevalence estimates [37].

The projected HIV prevalence and incidence for each country and in each arm are shown in Figure 3, demonstrating a good fit to the UNAIDS national prevalence estimates used for calibration. The saw-tooth pattern in incidence in the intervention arms projections reflects the six-monthly rounds of the intervention. In a sensitivity analysis, we found that rounds of 6 months for the CHiPs intervention would be preferable to rounds of 9 or 12 months, as they would lead to a greater reduction in HIV incidence over 3 years (see supporting information, in particular Figure S7 in File S1). The predicted relative reductions in HIV incidence for both countries are shown in Table 2. Under the central target, we estimated a reduction in 3-year cumulative incidence of 61% (Zambia) and 62% (South Africa) in arm A and 25% (Zambia) and 26% (South Africa) in arm B respectively, compared to standard of care (arm C), with an effect increasing from one year to the next.

**Table 2:** Projected impact of the intervention on HIV incidence in Arms A and B compared with Arm C for central and optimistic target scenarios (specific parameter values for each scenario defined in Table S10 in File S1).

Country	Relative reduction in…	Central target		Optimistic target	
		Arm A	Arm B	Arm A	Arm B
Zambia	3-year cumulative incidence	61%	25%	63%	27%
	2-year cumulative incidence	58%	24%	61%	25%
	First year incidence	51%	20%	54%	21%
	Second year incidence	65%	27%	67%	28%
	Third year incidence	67%	29%	68%	30%
South Africa	3-year cumulative incidence	62%	26%	64%	27%
	2-year cumulative incidence	59%	25%	61%	26%
	First year incidence	52%	22%	55%	23%
	Second year incidence	65%	28%	67%	29%
	Third year incidence	68%	29%	69%	30%

These results were based on parameter values that yielded HIV epidemics most closely matching UNAIDS prevalence estimates. Exploring a variety of parameter sets which fitted those relatively well, we found that very different combinations of parameter values relating to the contact structure in the population could match the prevalence data (see Figures S3 to S5 in File S1). This suggests that these data alone are not very informative about the structure of contacts between the three risk groups, or the characteristics of those groups.

Despite those differences, we found that the predicted reduction in HIV incidence over three years was relatively stable regardless of the parameter set chosen (see Figure S6 in File S1).

**Figure 4:**
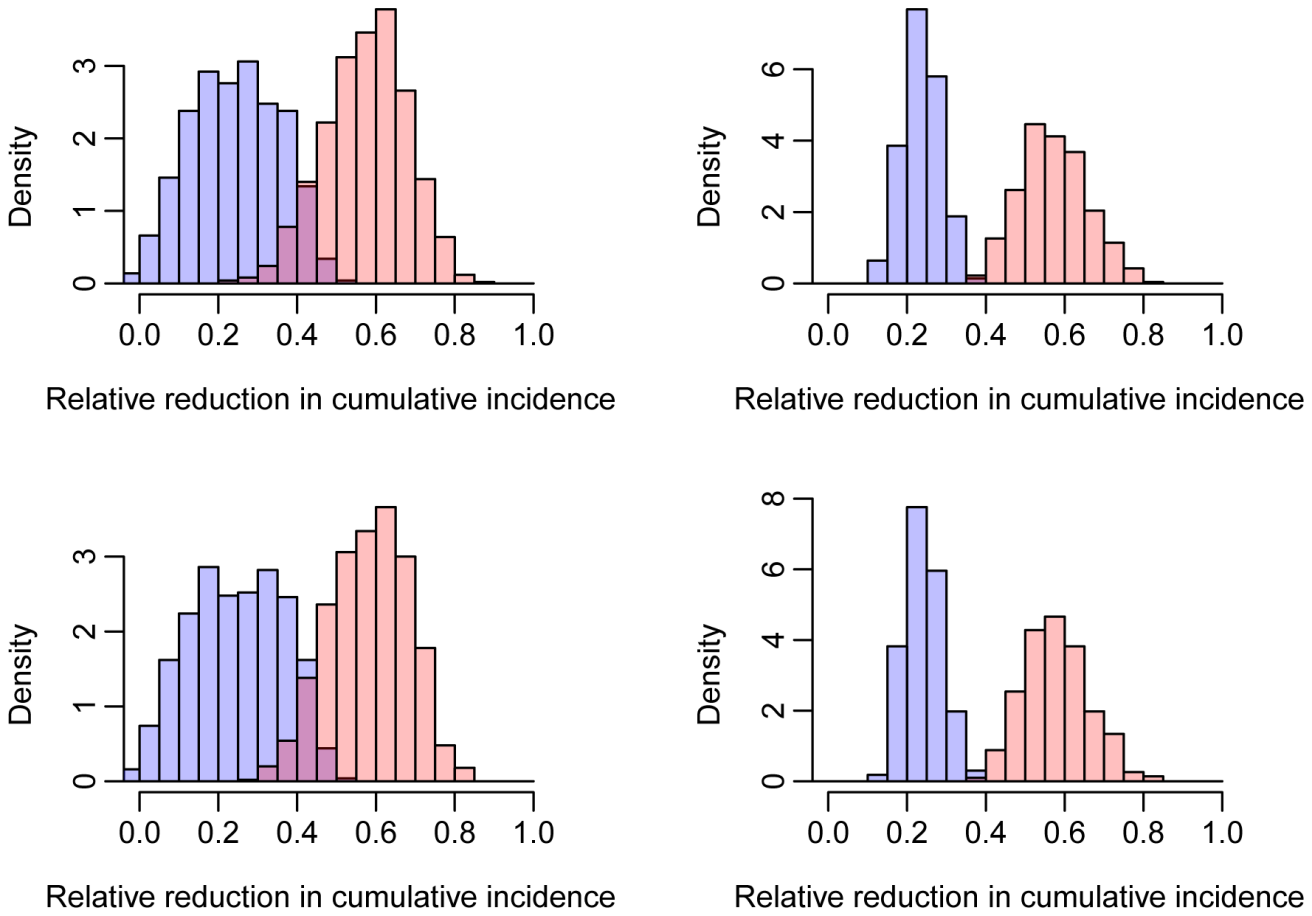
Uncertainty on the trial outcome in Zambia (top panels) and South Africa (bottom panels). The red and blue histograms show the relative reduction in 3-year cumulative incidence in arms A and B respectively when parameters vary within ranges shown in Table 1. The left panels show results obtained when all parameters are varied, and the right panels when assuming no population-level behavioural changes associated with the intervention.

However, the reduction in 3-year cumulative incidence was highly dependent on the value of process parameters such as the uptake of circumcision or testing, as illustrated in Figure 4. We found a very strong linear dependence of the relative reduction in 3-year cumulative HIV incidence on process parameters (adjusted R-squared >97% in both arms and both countries). The coefficients of the regression were strikingly similar between countries, although interestingly, a stronger influence of parameters related to circumcision was found in Zambia, where we assumed only 13% of men are circumcised during adolescence, than in South Africa, where we assumed 76% of men are circumcised during adolescence, as measured in ZAMSTAR [38], [39] (see Table 1).

Unsurprisingly, the major driver of the variability in the reduction in incidence was the magnitude of community-level changes in sexual risk behavior in response to the intervention activities (*i_bc_*), especially in arm B, where over 80% of the variance in the outcome is explained by *i_bc_*. We emphasize that in the model, behavior change refers to an overall response in the community, not to specific responses in individuals following ART. While this behavior is an overall modifier that affects all individuals, it will be balanced by counseling and treatment in those aware of their status. Therefore the principal manner in which this community change affects incidence is through changed risk behaviors in individuals who do not know their serostatus. In particular, changes towards more risky behaviors could jeopardize the success of the trial and lead to an increase in incidence in intervention arms compared to the control arm. On the other hand, protective changes in sexual behavior resulting from the trial activities could increase the reduction in incidence beyond that directly associated with the home-based testing, active linkage to care, ART and circumcision interventions. However, based on previous experience, we do not expect major changes in risk behavior during this trial, and our baseline scenarios reflect this assumption [41]–[43].

The second driver of the variability in the reduction in cumulative incidence was the relative infectivity of individuals under ART, which accounted for 34% (Zambia) and 35% (South Africa) of the variance in cumulative incidence in arm A, and approximately 8% in arm B in both countries. In fact, in arm A, the relative infectivity of individuals on ART was as important as the community-level changes in sexual risk behavior in response to the intervention activities, which accounted for 33% (Zambia) and 34% (South Africa) of the variability in cumulative incidence.

Other important drivers included the uptake of testing and ART, the proportion of sex acts with partners from outside of the community and the rate of drop-out from ART. The uptake of circumcision was found to have little influence on the outcome in South Africa, but a larger influence in Zambia, especially in arm B.

Although the intervention is planned to run for a 3-year duration, we looked at the impact of an intervention extended to a 10-year horizon under the central target. We found a reduction in 10-year cumulative incidence of 63% and 29% in arms A and B respectively in Zambia, and 64% and 29% in South Africa, compared to standard of care (arm C). The relative reduction in incidence for year 10 only would be 61% and 30% for arms A and B respectively in Zambia, and 64% and 31% in South Africa.

## Discussion

We developed a deterministic compartmental model to predict the potential impact of the intervention activities that will be undertaken in the HPTN 071 (PopART) trial, designed to explore the potential population effect of universal home-based testing (in arm B) and universal testing and treating (in arm A) on HIV incidence in large communities in Zambia and South Africa. Our pre-trial modeling analysis predicts that if intervention targets are reached, HIV incidence will decrease dramatically in both intervention arms, with a 3-year cumulative reduction of 61 to 62% in arm A and 25 to 26% in arm B, relative to standard of care (arm C), in both countries. Our model predicts that the reduction in cumulative HIV incidence associated with home-based HIV testing (>25% over 3 years in arm B) will be much greater than that effected by community-based HIV testing in a recent trial in sub-Saharan Africa and Thailand (14% over 3 years, not statistically significant) [44].

In addition to projecting the overall impact of the complex intervention package for the purposes of designing and ensuring adequate power in the trial, understanding, in this pre-trial phase, the modifiable factors affecting the reduction in incidence is crucial to prioritize allocation of human and financial resources to areas where the success of the intervention could be threatened. We showed that the reduction in 3-year cumulative incidence in the intervention arms compared to the control arm is almost linearly determined by a handful of process parameters. This linear dependency suggests that monitoring a few parameters during the course of the HPTN 071 (PopART), and other similar trials, should be enough to assess its progress in real time and to increase targeted efforts if needed.

Unsurprisingly, we found that important threats to the trial success would be increases in risky sexual behaviors at the population level in response to the trial activities and secondarily the uptake of testing and ART as well as non-adherence to treatment. We hypothesize that continued counseling, facilitated by the annual visits of the CHiP teams in all households will be important to prevent increased risk behaviors and promote adherence.

The uptake of circumcision appeared to be a relatively important factor in determining changes in incidence in Zambia, where the current circumcision levels are low, but less so in the South African trial sites, located in the Western Cape region where circumcision is more common.

We found that once uptake of the intervention and adherence are ensured, minimizing delays in linkage to care would favor the trial success, but might not be as crucial as could have been anticipated, if those delays do not greatly exceed those we have explored here.

Importantly, 10-year model projections suggested that in the long term, prolonged interventions similar to those proposed in arms A and B would allow to maintain HIV incidence at lower levels, but not achieve elimination. This result is different to that of Granich et al. [4] who found that annual incidence could be reduced below 1 per thousand per year within only a few years. However, that model made much more optimistic assumptions about the reduction in infectiousness for persons on ART (99% versus 90% in our central target scenario) as well as uptake of universal testing and treatment (92% of untreated persons per year versus 70% in our central target scenario), which we showed were important determinants of the reduction in HIV incidence. Our model predictions had previously been compared to predictions of eleven other models, in a study designed to assess the influence of assumptions regarding HIV epidemiology on the predicted impact of ART on HIV incidence [16]. Although long-term predictions varied substantially across models, our model was generally consistent with others, and rather conservative with regards to the long-term reductions in incidence due to ART. Epidemiologic and service uptake data collected during the trial, in conjunction with mathematical modelling, should improve the accuracy and precision of future model projections and allow re-evaluation of the effort required to achieve HIV elimination.

In the uncertainty analysis, we found that the relative reduction in incidence over the three years of the trial was largely insensitive to input parameters (such as structure of the sexual mixing matrix), despite great uncertainty on some of these parameters. However, such uncertainty can affect the long term projections of interventions, as illustrated by the variability in the predicted reduction in cumulative 10-year incidence in Arm A in South Africa (see Figure S6 in File S1) when assuming that the intervention were extended to a 10-year horizon. Similarly, improving the mechanistic representation of viral suppression, CD4 cell dynamics, and survival on ART, would probably affect the long-term projections, although unlikely to change the short-term ones. Data collected during the trial, in particular through the questionnaires administered in the population cohort and in planned case-control studies, will help quantify some of those parameters directly, notably the parameters describing the structure of contacts between and within risk groups. The prospect of combining these granular survey and biomarker data with novel phylogenetic methods for associating clusters of transmissions provides a unique opportunity to answer long-standing epidemiological questions, such as the amount of transmission occurring during primary HIV infection [45], patterns of sexual mixing, the geographic patterns of infection [46], [47] or the importance of core groups of highly transmissible or particularly at-risk individuals. This will be crucial when trying to generalize the trial results to wider spatial and temporal scales.

Indeed, the HPTN 071 (PopART) trial will be performed at an unusually large scale, with approximately 1.2 million individuals across all clusters and trial arms [11], [14]. Therefore this intervention could serve as a paradigm for routine implementation of universal testing and treatment on a provincial or even national scale. The economic analysis of HPTN 071 will provide guidance on whether routine implementation is a valuable investment in the health of populations, and is therefore an integral part of the trial. It will help policymakers assess the costs and benefits of universal testing and treatment, in comparison to alternative strategies. In a collaborative effort to estimate the cost-effectiveness of earlier ART eligibility and expanded access to ART in low- and middle-income settings, both the population-level health-benefits and particularly the implementation costs of earlier ART eligibility and achieving high levels of access to early ART were identified as key uncertainties and sources for caution in policy setting; the HPTN 071 trial is ideally placed to answer these questions. Many expected benefits of HPTN 071, including saved future healthcare costs due to secondary infections averted, will occur after the trial. It is therefore crucial to integrate the projected outcomes from the epidemiological model into an economic model, with the objective of calculating the cost-effectiveness of HPTN 071 over different time horizons.

The model used in this analysis relies on simplified representations of the complex dynamics of HIV infection and the determinants of the spread of HIV. Data collected during the trial will allow assessing the extent to which simplifications we have made, such as omitting age structure, considering only heterosexual sex, disregarding the nature of sex, assuming similar distribution of men and women amongst risk groups, or assuming independence between risk group and propensity to have sexual contacts outside of the community, are reasonable. The model also does not include selection and transmission of drug resistant strains of virus. A more detailed model, informed by those data, will be developed during the trial and used to help interpreting the trial results. This future model will also be able to account for how potential changes in the national treatment guidelines in Zambia and South Africa and other secular changes in the epidemic and the response to the epidemic affect the outcomes of the trial and the course of these severe HIV epidemics.

Our analysis highlights the role that mathematical modeling can play in trial development and monitoring, and more widely in evaluating the impact of treatment as prevention. In the case of the HPTN 071 (PopART) trial, we showed that a reduction in 3-year cumulative incidence by over 60% could be expected, but would require careful real-time monitoring of the intervention uptake to ensure adequate program coverage.

## Supporting Information

File S1 **Combined supporting information.** This file contains a detailed description of the model structure and parameterization as well as sensitivity analyses.
(PDF)
