# Supplementary material for: HPTN 071 (PopART): A Cluster-Randomized Trial of the Population Impact of an HIV Combination Prevention Intervention Including Universal Testing and Treatment: Mathematical Model
Source: PLoS One. 2014 Jan 15;9(1):e84511. doi: 10.1371/journal.pone.0084511 (PMC3893126; doi:10.1371/journal.pone.0084511)
Supplement: File S1 — Combined supporting information. This file contains a detailed description of the model structure and parameterization as well as sensitivity analyses. (PDF) [file pone.0084511.s001.pdf]

Supporting information for

HPTN 071 (PopART): a cluster-randomized trial of the population impact of an HIV combination prevention intervention including universal testing and treatment:
Mathematical model

Anne Cori[1], Helen Ayles[2,3], Nulda Beyers[4], Ab Schaap[2,5], Sian Floyd[5], Kalpana Sabapathy[5], Jeffrey W. Eaton[1], Katharina Hauck[6], Peter Smith[6], Sam Griffith[7], Ayana Moore[7], Deborah Donnell[8], Sten H. Vermund[9], Sarah Fidler[10], Richard Hayes[5], Christophe Fraser[1*] and the HPTN 071 (PopART) study team.

[1]MRC Centre for Outbreak Analysis and Modelling, Department of Infectious Disease Epidemiology, Imperial College London, London, UK.

[2] ZAMBART, University of Zambia, School of Medicine, Ridgeway Campus, Lusaka, Zambia

[3]Department of Clinical Research, London School of Hygiene and Tropical Medicine, London, UK

[4] Desmond Tutu TB Centre, Department of Paediatrics and Child Health, Stellenbosch University, South Africa

[5] Department of Infectious Disease Epidemiology, London School of Hygiene & Tropical Medicine, London, UK

[6] Business School, Imperial College London, South Kensington, London, UK

[7] FHI 360, Research Triangle Park, North Carolina, USA

[8] Vaccine and Infectious Disease Division, Fred Hutchinson Cancer Research Center, Seattle, Washington, USA

[9]Vanderbilt Institute for Global Health and Department of Pediatrics, Vanderbilt University, Nashville, Tennessee, USA

[10]Department of Medicine, Imperial College London, UK

[*] Corresponding author: Professor Christophe Fraser, MRC Centre for Outbreak Analysis and Modelling, Department of Infectious Disease Epidemiology, School of Public Health, Imperial College London, St Mary's Campus, Norfolk Place, London W2 1PG, UK. Tel: +44 20 7594 3397. Fax: +44 20 7402 3927. Email: c.fraser@imperial.ac.uk.

Acronyms
ART = Antiretroviral therapy
PopART = Population Effects of Antiretroviral Therapy to Reduce HIV Transmission
HBT = home-based voluntary testing
HPTN = HIV Prevention Trials Network
HPTN 071 = The HPTN study number for the PopART intervention
CD4 = CD4+ cell count per μL of peripheral blood
PMTCT = prevention of mother to child transmission of HIV

Notations
In the following,

$\mathbf{1}_A$ is the indicator function, with value $\mathbf{1}_A = 1$ if A is true and 0 otherwise.

$a \pmod{k}$ is the remainder of the Euclidian division of a by k.

$t_{start} = 2013.5$ is the date of start of the intervention.

Model structure
The model is a deterministic compartmental model describing the population aged 15 years and over, specified by a system of ordinary differential equations for the time-evolution of the number of individuals in different states. The time unit is the year.

Individuals are stratified according to sex (female/male), infection status (susceptible/infected), and sexual risk propensity (high/medium/low).

Susceptible males are classified as uncircumcised, uncircumcised waiting for circumcision, circumcised in healing period, or circumcised. Adult susceptible males who get circumcised go through each of those compartments sequentially. Susceptible males who are circumcised before the age of 15 enter directly the circumcised compartment. The proportion of men circumcised during adolescence ($mc_{birth} = 76\%$ in the Western Cape region of South Africa, 13% in Zambia) in each country was taken from data from the ZAMSTAR trial [1,2]. Males can be infected in any of these stages.

Infected individuals are classified as untreated, untreated waiting for treatment, treated but not virally suppressed, and treated and virally suppressed. Untreated individuals who engage into treatment go through these compartments sequentially. Those in the two later stages can drop out of treatment, coming back to the untreated compartment.

Infected individuals are further classified in one of five disease stages: the first one is the early (or acute) stage, followed by one of four stages, defined by the CD4 level (Stage 1 corresponds to CD4≥500, stage 2 to 350≤CD4<500, stage 3 to 200≤CD4<350, and stage 4 to CD4<200). Whilst for untreated individuals, the CD4 count referred to in this classification is the actual CD4 count of individuals, for those on ART, it refers to the CD4 count individuals would drop to should they interrupt treatment. The model of progression between these stages was matched to reflect a recent re-analysis of clinical cohort data [3] (see next section, Figure 2b and Table S 4).

Notations for the different compartments are described in Tables S 1 to S 3. The full flow diagram between the different compartments is shown in Figures S 1 (males) and S 2 (females).

Table S 1. "Social" characteristics

Characteristic	Notation	Detail
Sex	a	$a = f$: female<br>$a = m$: male<br>We define $\bar{a} = f$ if $a = m$ and $\bar{a} = m$ if $a = f$
Circumcision status (for males only)	x	$x = u$: uncircumcised<br>$x = p$: uncircumcised planning circumcision<br>$x = h$: circumcised healing<br>$x = c$: circumcised
Sexual behaviour	i	$i = 1$: high sexual activity<br>$i = 2$: moderate sexual activity<br>$i = 3$: low sexual activity
Localisation	*	Individuals living in neighbouring areas from the trial communities are indicated with a *

Table S 2. Infection stages

Characteristic	Notation	Detail
Infection stage	k	$k = 1$: CD4≥500<br>$k = 2$: 350≤CD4<500.<br>$k = 3$: 200≤CD4<350<br>$k = 4$: CD4<200<br>The acute infection is modelled by a separate state variable. For untreated individuals, the CD4 count referred to in this classification is the actual CD4 count of individuals; for those on ART, it refers to the CD4 count individuals would drop to should they interrupt treatment.

Table S 3. State variables. State variables represent the number of individuals in each state at different times during the epidemic. There are 288 state variables in total. Social characteristics are shown in subscripts (a, x and i refer to sex, circumcision status, and sexual behaviour respectively (see Table S1)). Infection stages are shown in superscripts (k, see Table S2). Individuals living in neighbouring areas from the trial communities are indicated with a *.

State variable	Notation
Susceptible (uninfected).	S_{axi} in the trial community;<br>S^*_{axi} in neighbouring areas
Infected, acute infection.	P_{mhi} ; P_{ai} ; P^*_{mhi} ; P^*_{ai}
Untreated chronic infection	I^k_{ai} ; I^{k*}_{ai}
Untreated chronic infection waiting for treatment	J^k_{ai} ; J^{k*}_{ai}
Treated chronic infection, not virally suppressed	T^k_{ai} ; T^{k*}_{ai}
Treated chronic infection, virally suppressed	A^k_{ai} ; A^{k*}_{ai}

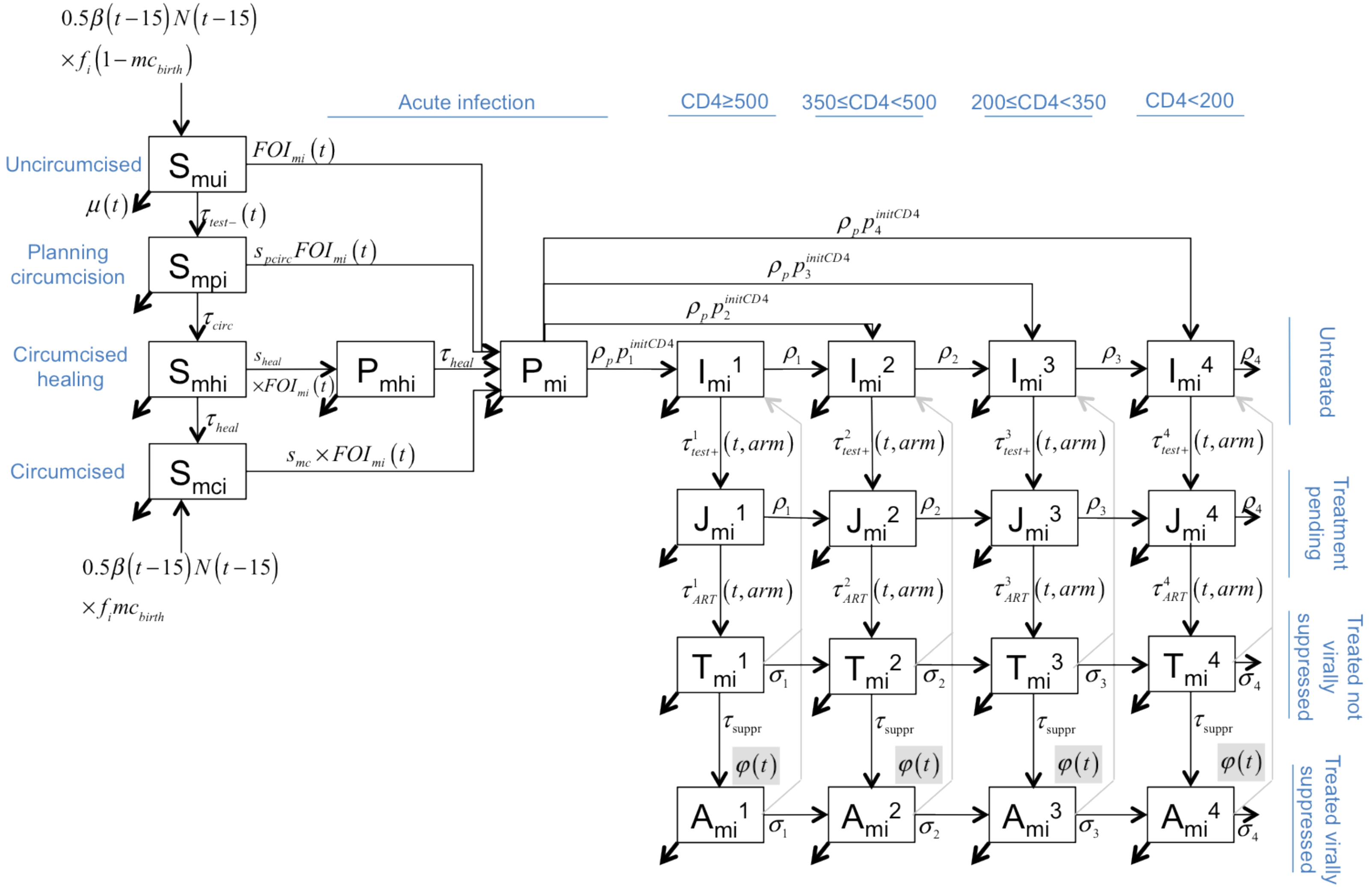

Figure S 1: Flow diagram for men in risk group i in the trial cluster. Other risk groups and neighbouring communities have similar flow diagrams. Arrows pointing to bottom left represent non-HIV related mortality.

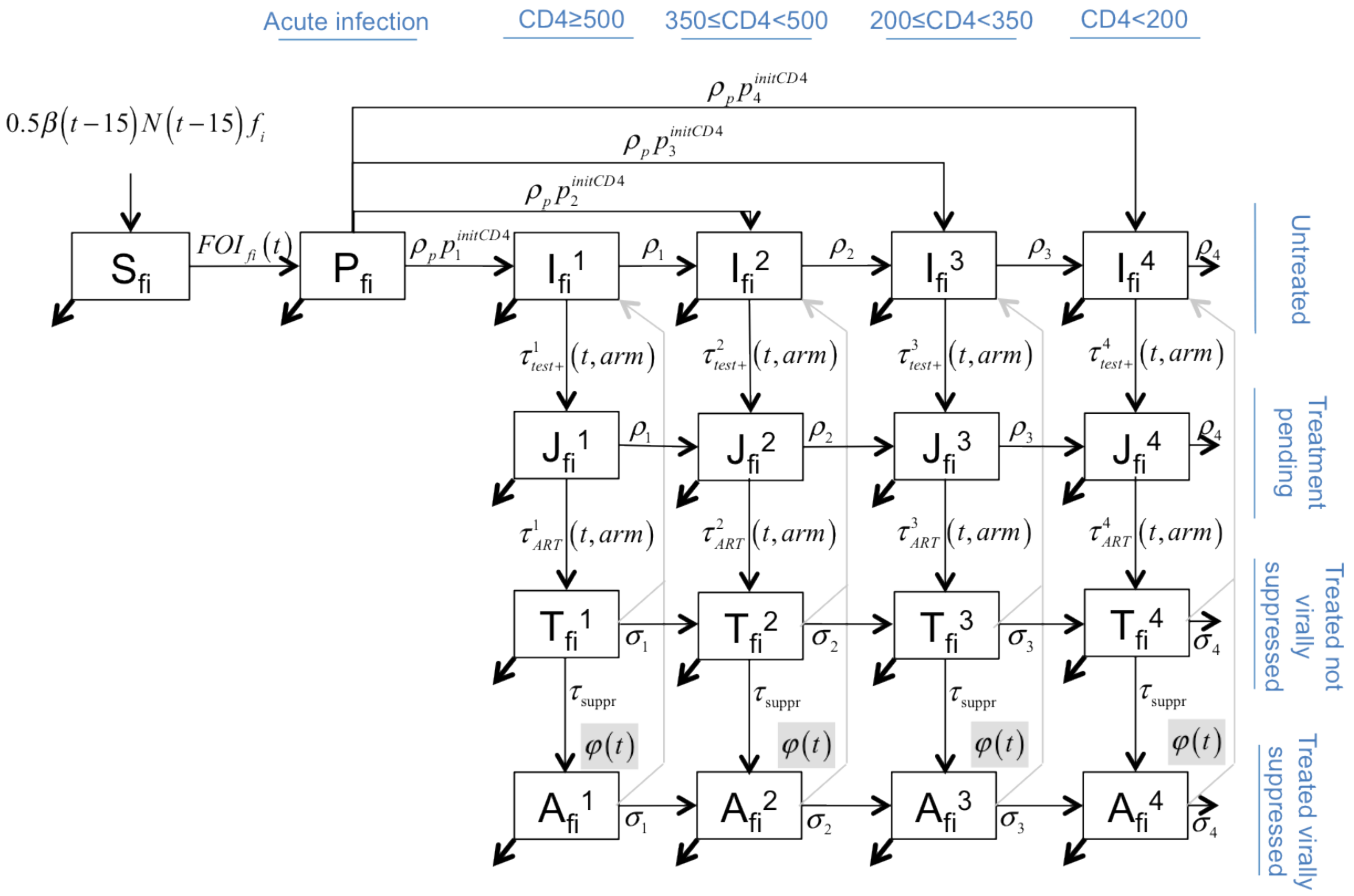

Figure S 2: Flow diagram for women in risk group i in the trial cluster. Other risk groups and neighbouring communities have similar flow diagrams. Arrows pointing to bottom left represent non-HIV related mortality.

Parameterizing disease progression

Upon becoming infected, all individuals first enter the acute infection stage, lasting for a mean of 0.24 years [4]. Following acute infection, individuals may enter any of the CD4 stages CD4 >500, CD4 350-500, CD4 200-350, and CD4 ≤200, and individuals progress to the next lowest stage at a constant rate. Individuals in the stage CD4 ≤200 experience HIV-related mortality. Parameters determining the proportion entering each CD4 cell count stage, $p_i^{initCD4}$, $i = 1,\dots,4$, and the progression rate between each stage, ρ_i, $i = 1,\dots,3$, were estimated by least-squares to obtain the closest fit to the percentage in each CD4 category annually for the first six years after seroconversion in recent model estimates based on European seroconverter cohorts (Figure 1 in [3]). The rate of HIV-related mortality for those with CD4 <200, ρ_4, was chosen to get an incidence of 35.2 deaths per 100 per year amongst those individuals [5]: $\rho_4 = -\ln(1-0.352) = 0.434$, leading to a mean survival from infection to death of 12.0 years (median 10.4 years).

For treated individuals, the model does not attempt to describe CD4 cell dynamics. Instead, the model captures in a simple stylised manner two important processes: the mortality of patients on ART and the CD4 level to which individuals drop when they interrupt treatment, which both depend mainly on the nadir CD4 count immediately prior to the initiation of ART and the time since ART initiation [6-10]. Here, we do not attempt to fit this part of our model to data. Instead, we use a similar simple assumption to Granich et al. [11], specifically that treated patients progress through nadir CD4 count categories at a rate σ_k which is half that of untreated patients.

Parameters relating to disease progression are summarized in Table S 4.

Table S 4. Clinical progression parameters. Subscript k shows the CD4 stage (see Table S 2).

Description	Symbol	Value	Ref
Rate of progression from acute to chronic infection (year^{-1})	ρ_p	4.14 (corresponding to mean duration of 2.9 months)	[4]
Rates of progression to the next chronic infection stage (year^{-1})	ρ_k	$\rho_1 = \frac{1}{6.37} = 0.157$<br>$\rho_2 = \frac{1}{2.86} = 0.350$<br>$\rho_3 = \frac{1}{3.54} = 0.282$<br>$\rho_4 = \frac{1}{2.30} = 0.434$	$k = 1,\dots,3$: Fitted to data from [3]<br>$k = 4$: adapted from [5]<br>Note that the mean survival time cannot be obtained from these alone since after primary infection individuals can directly enter low CD4 compartments.
Proportion starting in each CD4 class after acute infection	$p_k^{initCD4}$	$p_1^{initCD4} = 0.58$<br>$p_2^{initCD4} = 0.23$<br>$p_3^{initCD4} = 0.16$<br>$p_4^{initCD4} = 0.03$	Fitted to data from [3]
Rates of disease progression when under treatment (year^{-1})	σ_k	$\sigma_k = 0.5\rho_k$	[11] – see explanations for this counterintuitive assumption in the main text.

In a sensitivity analysis, we explored the impact of the intervention under the extreme scenario where $\sigma_k = 0$, that is, individuals on ART experience no HIV-related mortality, and

when they interrupt treatment, they return to the CD4 count they were at immediately before initiating treatment. The corresponding relative reduction in 3-year cumulative HIV incidence was 59% in arm A in both countries and 27% (Zambia) and 26% (South Africa) in arm B, compared to arm C. These results are very similar to those obtained in the main scenario we considered ($\sigma_k = 0.5\rho_k$), which confirms that the model of survival and CD4 cell dynamics on ART will not dramatically affect the short term projections. Interestingly, the 10-year projections were also relatively similar in both scenarios (60% (arm A) and 30 to 32% (arm B) reduction in 10-year cumulative HIV incidence in the scenario with $\sigma_k = 0$, compared to 64% (arm A) and 29% to 31% (arm B) in the scenario with $\sigma_k = 0.5\rho_k$).

Modelling testing, treatment and circumcision

We model separately a background process of HIV-related care, representing the current patterns of uptake of testing, treatment and circumcision services, and an additional process resulting from the intervention activities in the trial. Therefore the rates describing engagement into care are defined as the sum of a background term, and, for arms A and B and during the trial, an additional term describing the intervention. More precisely:

$$\tau_{test-}(t,arm) = \tau_{test-,background}(t) + \mathbf{1}_{\{arm \neq C\}}\mathbf{1}_{\{t>t_{start}\}}\tau_{test-,trial}(t)$$

$$\tau^k_{test+}(t,arm) = \tau^k_{test+,background}(t) + \mathbf{1}_{\{arm \neq C\}}\mathbf{1}_{\{t>t_{start}\}}\tau^k_{test+,trial}(t)$$

Background testing, treatment and circumcision

Background (i.e., not linked to the trial) treatment programmes (recruitment and drop-out) were adapted from Granich et al [11]. Background HIV testing is not modelled explicitly. Instead, we model the rate at which individuals initiate ART, which encompasses both testing and successful linkage to care. We assume that only individuals with CD4<350 can initiate treatment. The rate at which they do so is modelled as a Hill function increasing from 2004 onwards, with a greater rate for individuals with CD4<200.

More precisely, the rates at which untreated infected individuals enter the “awaiting for treatment” compartment is (depending on the CD4 category):

$$\tau^1_{test+,background}(t) = \tau^2_{test+,background}(t) = 0$$

$$\tau^3_{test+,background}(t) = \mathbf{1}_{\{t>2004\}}\tau_{\max}\frac{(t-2004)^{0.5}}{(t-2004)^{0.5}+10^{0.5}}$$

$$\tau^4_{test+,background}(t) = \mathbf{1}_{\{t>2004\}}2\tau_{\max}\frac{(t-2004)^{0.5}}{(t-2004)^{0.5}+10^{0.5}}$$

That is, prior to the trial as well as in arm C during the trial, there is no treatment initiation from CD4≥500 (τ^1) and CD4 350-500 (τ^2) stages, and individuals with CD4<200 link to ART at twice the rate of individuals with CD4 200-350 ($\tau^4 = 2\tau^3$) to allow for a faster treatment rate for individuals with CD4<200. The value of τ_{max} was constrained so that the total proportion on treatment in year 2010 matches data from the ZAMSTAR trial [1,2]; values for Zambia and South Africa were 0.513 and 0.305 year^{-1} respectively.

Regarding circumcision, we assume that, in all arms and both countries, a certain proportion of males are circumcised prior to entry into the modelled population at age 15. We assume

these are fully circumcised. We do not model any adult circumcision outside of that offered as part of the intervention package in arms A and B:

$$\tau_{test-,background}(t)=0.$$

Additional testing, treatment and circumcision in arms A and B during the trial

In addition to this background process, during the trial, community HIV-care providers (CHiPs) teams will offer, in arms A and B, home-based testing every year during 6-months rounds in the intervention arms of the trial. These intervention rounds are scheduled to last 6 months: here we model these taking place from 1st July to 31st December, from 2013 to 2015. These annual rounds of testing are modelled by a constant number of tests offered each day during those time periods by CHiPs.

Following testing by CHIPS (which, when offered, is only accepted by a proportion p_{test} of individuals), a fraction p_{circ} of susceptible men who have accepted testing will decide to get circumcised and a fraction p_{ART} of infected individuals who have accepted testing will decide to get treated.

Parameterization of the rate of HIV testing by CHiPs was not straightforward since, generally speaking, a constant flow out of a compartment *X* (here, a compartment of individuals who receive HIV testing), of size $X(t)$, cannot be modelled by a constant rate τ (here, rate of HIV testing). To get a constant flow, the rate has to be parameterized as $\tau(t)=\tau(t_0)\frac{X(t_0)}{X(t)}$ so that the flow is constant: $\tau(t)X(t)=\tau(t_0)X(t_0)$ (t_0 being an arbitrary date). This parameterization leads to a linear decrease in $X(t)$. However, if there are other routes out of the compartment *X* (such as infection, migrations, deaths), this can lead to negative value of $X(t)$. In our model, this reflects for instance the fact that individuals who have died (or initiated treatment outside of the trial context) before the CHiPs rounds will not be offered a test.

Therefore the rate has to be multiplied by the proportion of those expected to be in *X* at time *t* who are actually still in compartment *X* at time *t*: $\tau(t)=\tau(t_0)\frac{X(t_0)}{X(t)}\frac{X(t)}{X(t_0)\left(1-\tau(t_0)\times(t-t_0)\right)}$, which simplifies to $\tau(t)=\frac{\tau(t_0)}{1-\tau(t_0)(t-t_0)}$.

Therefore, overall, the rate of susceptible men entering the waiting circumcision stage is:

$$\tau_{test-,trial}(t)=\frac{\tau_{testCHIPS}(t)p_{test}p_{circ}}{1-\tau_{testCHIPS}(t)p_{test}p_{circ}\times\left[(t-t_{start})(\text{mod}1)\right]}$$

and the rate of untreated infected individuals entering the waiting for treatment stage is:

$$\tau_{test+,trial}(t)=\frac{\tau_{testCHIPS}(t)p_{test}p_{ART}}{1-\tau_{testCHIPS}(t)p_{test}p_{ART}\times\left[(t-t_{start})(\text{mod}1)\right]}$$

with $\tau_{testCHIPS}(t) = \mathbf{1}_{\{t > t_{start}\}} \mathbf{1}_{\{(t - t_{start})(\mathrm{mod}\,1) \le D\}} \frac{1}{D}$; D = 0.5 year the duration of the CHiPs rounds each year; $\mathbf{1}_{\{(t - t_{start})(\mathrm{mod}\,1) \le D\}}$ equals 1 when t is during the last six months of each calendar year (where the intervention takes place) and zero during the rest of the year; and $(t - t_{start})(\mathrm{mod}\,1)$ the time since the start of the latest round of intervention.

Individuals who accept testing and circumcision or treatment then enter the "waiting" stages before actually getting circumcised, at a rate τ_{circ} or treated at a rate τ^{k}_{ART} dependent on disease stage k. $\frac{1}{\tau^{k}_{ART}}$ reflects the average time from testing to ART initiation. In arm A, this encompasses the time for individuals to show up at a clinic, get a CD4 test, and start treatment irrespective of CD4 count: $\tau^{1}_{ART}(t) = \tau^{2}_{ART}(t) = \tau^{3}_{ART}(t) = \tau^{4}_{ART}(t) = \frac{1}{\nu_{wait}(t)}$, with ν_{wait} the average time between HIV testing and treatment initiation, which encompasses the delays between testing and visit to the clinic, the delay between visit to the clinic and CD4 test results, and the delay between CD4 test results and actual ART initiation when eligible. In arms B and C (as well as before the intervention and in the neighbouring communities), this additionally accounts for the fact that non-eligible individuals will not start treatment: $\tau^{1}_{ART}(t) = \tau^{2}_{ART}(t) = 0; \quad \tau^{3}_{ART}(t) = \tau^{4}_{ART}(t) = \frac{1}{\nu_{wait}(t)}$.

With this parameterization, in arm B, individuals who are tested at high CD4 counts remain in the "waiting treatment" stage until they reach CD4<350, after which they initiate treatment at a rate $\tau^{3}_{ART}(t) = \frac{1}{\nu_{wait}(t)}$, that is on average after 4 weeks. However, these individuals have to come back for repeated CD4 counts before finding out they are eligible for treatment. But a large proportion of patients do not come back for these tests [12], and if they do, it is at a much lower rate than individuals coming for their first CD4 count (national recommendation for pre-ART monitoring in Zambia and South Africa is once every 3 or 6 months depending on current CD4 count). To account for this effect, we assumed that in fact these individuals remained in the untreated compartments until they reached CD4<350 and were then revisited by CHiPS teams (i.e. $\tau^{1}_{test+,trial}(t, arm = B) = \tau^{2}_{test+,trial}(t, arm = B) = 0$). They could then enter the "waiting treatment" stage at a rate $\tau^{k}_{test+,trial}(t)$ (k=3 or 4).

Once circumcised, adult males go through a healing period that lasts on average $\frac{1}{\tau_{heal}}$ after which they are healed and circumcised. Similarly, after ART initiation individuals first enter a phase where they are not virally suppressed, which lasts on average $\frac{1}{\tau_{\mathrm{suppr}}}$ after which they are finally virally suppressed.

Treatment failure and drop-out

Individuals on ART can drop out (or have treatment failure). They are then assumed to go back to the “untreated” stage. They may then be re-started on ART at a later time. The rates of recruitment into care are the same for individuals who are recruited for the first time and others. We assume a fixed annual drop-out rate φ_0, which can be expressed as $\varphi_0 = 1 - e^{-\int_0^1 \varphi dt} = 1 - e^{-\varphi}$, where φ is the instantaneous drop-out rate. Therefore $\varphi = -\ln(1-\varphi_0)$. In a sensitivity analysis, we allow the annual drop-out rate to change to a new value φ_{UTT} in the communities receiving the intervention. The corresponding instantaneous drop-out rate is then calculated with a similar formula.

Parameters related to testing, circumcision and treatment are described in Table S 5.

Table S 5. Model parameters related to testing, circumcision and treatment.

Description	Symbol	Value	Ref
**Testing and circumcision combined**			
Rate of testing and deciding to get circumcised	$\tau_{test-}(t,arm)$	$\tau_{test-,background}(t)+\mathbf{1}_{\{arm\neq C\}}\mathbf{1}_{\{t>t_{start}\}}\tau_{test-,trial}(t)$	
Background rate of testing and deciding to get circumcised	$\tau_{test-,background}(t)$	0	
Intervention-related rate of testing and deciding to get circumcised	$\tau_{test-,trial}(t)$	$\frac{\tau_{testCHIPS}(t)p_{test}p_{circ}}{1-\tau_{testCHIPS}(t)p_{test}p_{circ}\times\left[(t-t_{start})(\mathrm{mod}1)\right]}$	For explanations on this ratio see page 8.
**Testing and treatment combined**			
Rate of testing and deciding to get treated	$\tau^{k}_{test+}(t,arm)$	$\tau^{k}_{test+,background}(t)+\mathbf{1}_{\{arm\neq C\}}\mathbf{1}_{\{t>t_{start}\}}\tau^{k}_{test+,trial}(t)$	
Background rate of testing and deciding to get treated	$\tau^{k}_{test+,background}(t)$	$\tau^{1}_{test+,background}(t)=\tau^{2}_{test+,background}(t)=0$ $\tau^{3}_{test+,background}(t)=\mathbf{1}_{\{t>2004\}}\tau_{\max}\frac{(t-2004)^{0.5}}{(t-2004)^{0.5}+10^{0.5}}$ $\tau^{4}_{test+,background}(t)=\mathbf{1}_{\{t>2004\}}2\tau_{\max}\frac{(t-2004)^{0.5}}{(t-2004)^{0.5}+10^{0.5}}$	
Intervention-related rate of testing and deciding to get treated	$\tau^{k}_{test+,trial}(t)$	$\tau^{k}_{test+,trial}(t,arm)=\mathbf{1}_{\{arm=A\text{ or }(arm=B\text{ and }k>2)\}}$ $\times\frac{\tau_{testCHIPS}(t)p_{test}p_{ART}}{1-\tau_{testCHIPS}(t)p_{test}p_{ART}\times\left[(t-t_{start})(\mathrm{mod}1)\right]}$	For explanations on this ratio see page 8.
Maximum background rate of testing and deciding to get treated (year^{-1})	$\tau_{\max}$	0.513 (Zambia) 0.305 (south Africa)	Fitted to data from the ZAMSTAR trial [1,2]

**Testing alone**			
Probability of accepting HIV test if offered by CHiPs	p_{test}	0.837 (central target)	[13,14]
Duration of CHiPs rounds (year)	D	0.5 year	Corresponds to 6 months
**Circumcision**			
Probability of getting circumcised given negative HIV test deliverd by CHiPs	p_{circ}	$p_{circ} = 0.5$ (central target)	
Rate of getting circumcised once decision to get circumcised has been made ($year^{-1}$)	τ_{circ}	$\tau_{circ} = 26$ (central target)	corresponds to an average time to get circumcised of 2 weeks
Rate of circumcision healing ($year^{-1}$)	τ_{heal}	$\tau_{heal} = 26$	corresponding to an average healing period of two weeks[15]
**Treatment**			
Probability of going to get treatment given a positive HIV test deliverd by CHiPs	p_{ART}	0.837 (central target)	[13]
Rate of treatment initiation after testing and deciding to get tested	$\tau^{k}_{ART}(t)$	$\tau^{1}_{ART}(t) = \tau^{2}_{ART}(t) = 0$ (before trial and during trial in arms B and C)<br>$\tau^{3}_{ART}(t) = \tau^{4}_{ART}(t) = \frac{1}{\nu_{wait}(t)}$ (during trial in arm A)<br>$\tau^{3}_{ART}(t) = \tau^{4}_{ART}(t) = \frac{1}{\nu_{wait}(t)}$ (all arms)	
Mean time between positive test and treatment initiation when eligible (year)	ν_{wait}	$\frac{1}{13}$ (central target)	=4 weeks
Rate of viral suppression for individuals on ART ($year^{-1}$)	τ_{suppr}	τ_{suppr} =8	Corresponds to a mean time to suppression of 1.5 months, based on [16]
Annual drop-out rate when no intervention	φ_0	$\varphi_0 = 0.1$	
Annual drop-out rate with intervention	φ_{UTT}	$\varphi_{UTT} = 0.1$ (central target)	
Instantaneous drop-out rate	φ	$\varphi = -\ln(1-\varphi_0)$ in absence of intervention<br>$\varphi = -\ln(1-\varphi_{UTT})$ in presence of intervention	

Contact patterns, relative susceptibility and relative infectivity

We use a model of assortative heterosexual sexual mixing between three sexual risk groups. The risk groups are defined by decreasing rates of partner change: $c_1 > c_2 = 1 > c_3 = 0.1$. The proportion f_i of the population in each group i is fitted. We assume partnerships are made preferentially within the same risk group, with a level of assortativity θ which is calibrated to fit prevalence estimates [17]. We further assume that within a partnership, unprotected sexual acts occur at an instantaneous rate which depends on the risk groups of the two individuals: it is the same for all partnerships between individuals of different risk groups, as well as partnerships between two mid-risk individuals; it is twice higher for partnerships between two high-risk individuals ($\psi_1 = 2$) and twice lower for partnerships between two low-risk individuals ($\psi_3 = 0.5$) [18].

To allow for contamination, we assumed that $\pi = 5\%$ (in the central target scenario) of partnerships are formed with partners from outside the study community. This value is varied in sensitivity analyses.

Parameters relating to risk groups and contacts are shown in Table S 6.

Table S 6. Parameters relating to risk groups and contacts. Subscript i shows the risk group (see Table S 1).

Description	Symbol	Value
Rate of partner change	c_i	$c_1 > 1$ is fitted (see Table S7)<br>$c_2 = 1$<br>$c_3 = 0.1$
Proportion in each risk group	f_i	Fitted (see Table S7)
Assortativity	θ	Fitted (see Table S7)
Relative rate of unprotected sexual acts within same-risk group partnerships[1]	ψ_i	$\psi_1 = 2$<br>$\psi_2 = 1$<br>$\psi_3 = 0.5$
Proportion of sexual acts with partners outside of the community	π	$\pi = 5\%$ (central target)

[1]relative to partnerships between individuals of different risk groups

Susceptibility is assumed to be decreased by circumcision, and infectivity to be greater during early/acute and late stage infection, and reduced in individuals on ART (but less so for non-virally suppressed individuals). Men in the wound healing period after circumcision are assumed to have a decreased sexual activity, but an increased susceptibility per sex act, leading to an overall reduced susceptibility. Similarly, if infected during the healing period, men are assumed to be more infectious until the end of the healing period, but accounting for their decreased sexual activity, they are overall less infectious.

In a sensitivity analysis, we also investigate the possibility that individuals in the "waiting" stages benefit from the HIV counselling delivered with the test and therefore reduce their unprotected sexual activity, resulting in lower susceptibility and infectivity levels. We also allow possible changes in behaviours at the community level (*i.e.,* in both infected and uninfected individuals) associated with the intervention leading to an overall change in the sexual contact rate by a factor i_{bc}.

Parameters relating to relative susceptibility and infectivity are shown in Table S 7. Relative susceptibility and infectivity of different stages are also summarised in Figure 1b and Figure 2c.

Table S 7. Susceptibility and infectivity of different stages. Subscript k shows the CD4 stage (see Table S 2).

Description	Symbol	Value	Ref
**Basic transmission rate**, defined as the rate at which an untreated infected individual with CD4≥350 not in acute infection transmits to a partner, assuming they are both in the mid-risk group	λ_0	Fitted (see Table S7)	
**Relative infectivity[1] of**			
Acute infection	i_p	26.04	adapted from [4]
Each chronic infection stage	i_k	$i_1 = i_2 = i_3 = 1$<br>$i_4 = 2.34$	adapted from [4]
Infected men in circumcision healing period	i_{heal}	$3.5 \times 0.11 = 0.385$	[19,20]
Individuals waiting treatment	i_{pART}	1 (central target)	
Individuals on ART not virally suppressed	i_{ART0}	0.5	
Individuals on ART virally suppressed	i_{ART}	0.1 (central target)	[21]
All infected individuals due to behavioural changes associated with the intervention	i_{bc}	1 (central target)	
**Relative susceptibility[2] of**			
Circumcised men	s_{mc}	0.4	[22-25]
Men in circumcision healing period	s_{heal}	$3 \times 0.11 = 0.33$	[20]
Men waiting circumcision	s_{pcirc}	1 (central target)	

[1]relative to uncircumcised untreated males with 200≤CD4<500, [2] relative to uncircumcised males

Demography

In order to realistically describe the demographic dynamics in each country, we used time varying per-adult birth rates $\beta(t)$ and adult population sizes $N(t)$ taken from the literature (passed and future projections, see below) as inputs to the model (where adults are defined as individuals aged over 15).

The non-HIV related death rate $\mu(t)$ was calculated dynamically so that the population size resulting from the model would match that from the literature:

$$\mu(t) = \frac{1}{N(t)}\left[\beta(t-15)N(t-15) - \rho_4 \sum_{i=1}^{3}\left(I_{fi}^4 + J_{fi}^4 + I_{mi}^4 + J_{mi}^4\right)(t) - \sigma_4 \sum_{i=1}^{3}\left(T_{fi}^4 + A_{fi}^4 + T_{mi}^4 + A_{mi}^4\right)(t) - \frac{N(t+\Delta t) - N(t)}{\Delta t}\right]$$

with $\Delta t = \frac{1}{104}$ year (corresponding to half a week) the time step of resolution of the system of differential equations.

We assumed that the projected birth rates and population sizes taken from the literature represented projections under a scenario without interventions (i.e. arm C). We run the model

under this scenario, and calculated the corresponding death rates. These death rates were then used as inputs for simulations run with interventions (i.e., arms A and B). This way, we assumed the same birth and death rates for all scenarios, but allowed varying population sizes according to the different scenarios. As a result, the population size in arm C was that taken from the literature, and the population sizes in arms A and B were larger because of reduced HIV-related deaths.

The adult population sizes $N(t)$ and per capita birth rate $\beta_0(t)$ from mid-1978 onwards were taken from http://www.un.org/esa/population/. The per capita birth rates were then divided by a constant κ representing the proportion of adults in the population ($\kappa = 0.53$ for Zambia and $\kappa = 0.70$ for South Africa, same source) to obtain the per-adult birth rates $\beta(t) = \frac{\beta_0(t)}{\kappa}$.

The rate of sexual maturation was assumed a fixed 15 years, and we neglected child mortality, so that the rate of new entrants into the sexually active class was number of births 15 years ago. We assumed half of births were of each sex.

Demographic parameters are shown in Table S 8.

Table S 8. Demographic parameters.

Description	Symbol	Value	Ref
Per-adult birth rate[1]	$\beta(t)$	$\beta(t) = \frac{\beta_0(t)}{\kappa}$	
Per capita birth rate	$\beta_0(t)$		http://www.un.org/esa/population/
Proportion of adults in the population[1]	κ	$\kappa = 0.53$ (Zambia) and $\kappa = 0.70$ (South Africa)	http://www.un.org/esa/population/
Non HIV-related death rate	$\mu(t)$	Calculated so that model run in arm C produces population sizes consistent with projections from literature	
Total adult population size[1]	$N(t)$		http://www.un.org/esa/population/

[1]adults defined as aged 15 and over.

Equations for the trial clusters

The following system of differential equations describes the dynamics of the number of individuals in each state: susceptible (1), acute infection (2), chronic/untreated infection (3), infected/waiting for treatment (4), infected/treated but not virally suppressed (5) and infected/treated and virally suppressed (6). State variables are defined in Table S 3 and parameters in Table S 4 to S 8. The epidemic in the cluster and in the neighbouring communities is seeded with 0.01% of the population, all males in acute infection, allocated to the three risk groups proportionally to their size. The time t_0 of seeding is fitted.

$$\left\{\begin{array}{l}
\frac{dS_{mui}}{dt} = \beta\left(t-15\right)N\left(t-15\right)0.5f_i\left(1-mc_{birth}\right)-\mu S_{mui}-FOI_{mi}S_{mui}-\tau_{test-}S_{mui} \\
\frac{dS_{mpi}}{dt} = \tau_{test-}S_{mui}-\mu S_{mpi}-s_{pcirc}FOI_{mi}S_{mpi}-\tau_{circ}S_{mpi} \\
\frac{dS_{mhi}}{dt} = \tau_{circ}S_{mpi}-\mu S_{mhi}-s_{heal}FOI_{mi}S_{mhi}-\tau_{heal}S_{mhi} \\
\frac{dS_{mci}}{dt} = \beta\left(t-15\right)N\left(t-15\right)0.5f_i mc_{birth}+\tau_{heal}S_{mhi}-\mu S_{mci}-s_{mc}FOI_{mi}S_{mci} \\
\frac{dS_{fi}}{dt} = \beta\left(t-15\right)N\left(t-15\right)0.5f_i-\mu S_{fi}-FOI_{fi}S_{fi}
\end{array}\right\}(1)$$

where FOI_{mi} and FOI_{fi} are the forces of infections applied to males and females in risk group *i* (see below).

$$\left\{\begin{array}{l}
\frac{dP_{mhi}}{dt} = FOI_{mi}s_{heal}S_{mhi}-\left(\mu+\tau_{heal}\right)P_{mhi} \\
\frac{dP_{mi}}{dt} = FOI_{mi}\left(S_{mui}+s_{pcirc}S_{mpi}+s_{mc}S_{mci}\right)+\tau_{heal}P_{mhi}-(\mu+\rho_p)P_{mi} \\
\frac{dP_{fi}}{dt} = FOI_{fi}S_{fi}-(\mu+\rho_p)P_{fi}
\end{array}\right\}(2)$$

$$\left\{\begin{array}{l}
\frac{dI_{ai}^1}{dt} = \rho_p p_1^{initCD4}P_{ai}-(\mu+\rho_1+\tau_{test+}^1)I_{ai}^1+\varphi\left(T_{ai}^1+A_{ai}^1\right) \\
\frac{dI_{ai}^k}{dt} = \rho_p p_k^{initCD4}P_{ai}+\rho_{k-1}I_{ai}^{k-1}-(\mu+\rho_k+\tau_{test+}^k)I_{ai}^k+\varphi\left(T_{ai}^k+A_{ai}^k\right) \text{ for } k=2,3,4
\end{array}\right\}(3)$$

$$\left\{\begin{array}{l}
\frac{dJ_{ai}^1}{dt} = \tau_{test+}^1 I_{ai}^1-(\mu+\rho_1+\tau_{ART}^1)J_{ai}^1 \\
\frac{dJ_{ai}^k}{dt} = \tau_{test+}^k I_{ai}^k+\rho_{k-1}J_{ai}^{k-1}-(\mu+\rho_k+\tau_{ART}^k)J_{ai}^k \text{ for } k=2,3,4
\end{array}\right\}(4)$$

$$\left\{\begin{array}{l}
\frac{dT_{ai}^1}{dt} = \tau_{ART}^1 J_{ai}^1-(\mu+\sigma_1+\tau_{\text{suppr}}+\varphi)T_{ai}^1 \\
\frac{dT_{ai}^k}{dt} = \tau_{ART}^k J_{ai}^k+\sigma_{k-1}T_{ai}^{k-1}-(\mu+\sigma_k+\tau_{\text{suppr}}+\varphi)T_{ai}^k \text{ for } k=2,3,4
\end{array}\right\}(5)$$

$$\left\{\begin{array}{l}
\frac{dA_{ai}^1}{dt} = \tau_{\text{suppr}}T_{ai}^1-(\mu+\sigma_1+\varphi)A_{ai}^1 \\
\frac{dA_{ai}^k}{dt} = \tau_{\text{suppr}}T_{ai}^k+\sigma_{k-1}A_{ai}^{k-1}-(\mu+\sigma_k+\varphi)A_{ai}^k \text{ for } k=2,3,4
\end{array}\right\}(6)$$

A proportion π of sex acts takes place with individuals from neighbouring areas in which the epidemic is simulated using the same model without the intervention (*i.e.* parameters for those are as in Arm C). Moreover, a proportion θ of sexual contacts are assumed to be formed assortatively—with members of the same sexual risk group—while the remaining $1-\theta$, are selected at random. Therefore the force of infection can be written as:

$$FOI_{ai} = c_i \left((1-\theta)\frac{\sum_{j=1}^{3} c_j K_{\bar{a}j}^{rand}}{\sum_{j=1}^{3} c_j N_{\bar{a}j}} + \theta \frac{K_{\bar{a}i}^{assort}}{N_{\bar{a}i}} \right)(1-\pi) + c_i \left((1-\theta)\frac{\sum_{j=1}^{3} c_j K_{\bar{a}j}^{rand*}}{\sum_{j=1}^{3} c_j N_{\bar{a}j}^{*}} + \theta \frac{K_{\bar{a}i}^{assort*}}{N_{\bar{a}i}^{*}} \right)\pi$$

In the formula above, the $K_{\bar{a}j}$s are the sum of all infected individuals of sex *a* and risk group *j*, weighted by their current transmission potential (accounting for relative infectivity of different stages), and rate of partner change, which depends on whether contact is assortative or not. For random mixing, partnerships are considered to be of intermediate length, that is on average $1/c_2$. We also account for the fact that individuals can change partners or move to a different stage of infection before infecting their current partner: therefore we consider the relative likelihood of infection compared to other possible events, which explains the ratios in the equations below (see [4,26] for detail):

$$K_{mi}^{rand} = \left(\begin{array}{l} c_2 \dfrac{\lambda i_p i_{heal}}{c_2 + \lambda i_p i_{heal} + \tau_{heal} + \mu} P_{mhi} + c_2 \dfrac{\lambda i_p}{c_2 + \lambda i_p + \rho_p + \mu} P_{mi} \\ + \sum_{k=1}^{4} c_2 \dfrac{\lambda i_k}{c_2 + \lambda i_k + \rho_k + \tau_{test+}^{k} + \mu} I_{mi}^{k} + \sum_{k=1}^{4} c_2 \dfrac{\lambda i_k}{c_2 + \lambda i_k + \rho_k + \tau_{ART}^{k} + \mu} J_{mi}^{k} \\ + \sum_{k=1}^{4} c_2 \dfrac{\lambda i_k i_{ART0}}{c_2 + \lambda i_k i_{ART0} + \sigma_k + \tau_{suppr} + \varphi + \mu} T_{mi}^{k} \\ + \sum_{k=1}^{4} c_2 \dfrac{\lambda i_k i_{ART}}{c_2 + \lambda i_k i_{ART} + \sigma_k + \varphi + \mu} A_{mi}^{k} \end{array} \right)$$

$$K_{fi}^{rand} = \left(\begin{array}{l} c_2 \dfrac{\lambda i_p}{c_2 + \lambda i_p + \rho_p + \mu} P_{fi} \\ + \sum_{k=1}^{4} c_2 \dfrac{\lambda i_k}{c_2 + \lambda i_k + \rho_k + \tau_{test+}^{k} + \mu} I_{fi}^{k} + \sum_{k=1}^{4} c_2 \dfrac{\lambda i_k}{c_2 + \lambda i_k + \rho_k + \tau_{ART}^{k} + \mu} J_{fi}^{k} \\ + \sum_{k=1}^{4} c_2 \dfrac{\lambda i_k i_{ART0}}{c_2 + \lambda i_k i_{ART0} + \sigma_k + \tau_{suppr} + \varphi + \mu} T_{fi}^{k} \\ + \sum_{k=1}^{4} c_2 \dfrac{\lambda i_k i_{ART}}{c_2 + \lambda i_k i_{ART} + \sigma_k + \varphi + \mu} A_{fi}^{k} \end{array} \right)$$

$$K_{mi}^{assort} = \left(\begin{array}{l} c_i \dfrac{\lambda \psi_i i_p i_{heal}}{c_i + \lambda \psi_i i_p i_{heal} + \tau_{heal} + \mu} P_{mhi} + c_i \dfrac{\lambda \psi_i i_p}{c_i + \lambda \psi_i i_p + \rho_p + \mu} P_{mi} \\ + \sum_{k=1}^{4} c_i \dfrac{\lambda \psi_i i_k}{c_i + \lambda \psi_i i_k + \rho_k + \tau_{test+}^{k} + \mu} I_{mi}^{k} + \sum_{k=1}^{4} c_i \dfrac{\lambda \psi_i i_k}{c_i + \lambda \psi_i i_k + \rho_k + \tau_{ART}^{k} + \mu} J_{mi}^{k} \\ + \sum_{k=1}^{4} c_i \dfrac{\lambda \psi_i i_k i_{ART0}}{c_i + \lambda \psi_i i_k i_{ART0} + \sigma_k + \tau_{suppr} + \varphi + \mu} T_{mi}^{k} \\ + \sum_{k=1}^{4} c_i \dfrac{\lambda \psi_i i_k i_{ART}}{c_i + \lambda \psi_i i_k i_{ART} + \sigma_k + \varphi + \mu} A_{mi}^{k} \end{array} \right)$$

$$K_{fi}^{assort}=\left(\begin{array}{l} c_i\dfrac{\lambda\psi_i i_p}{c_i+\lambda\psi_i i_p+\rho_p+\mu}P_{fi} \\ +\displaystyle\sum_{k=1}^{4}c_i\dfrac{\lambda\psi_i i_k}{c_i+\lambda\psi_i i_k+\rho_k+\tau_{test+}^k+\mu}I_{fi}^k+\sum_{k=1}^{4}c_i\dfrac{\lambda\psi_i i_k}{c_i+\lambda\psi_i i_k+\rho_k+\tau_{ART}^k+\mu}J_{fi}^k \\ +\displaystyle\sum_{k=1}^{4}c_i\dfrac{\lambda\psi_i i_k i_{ART0}}{c_i+\lambda\psi_i i_k i_{ART0}+\sigma_k+\tau_{\text{suppr}}+\varphi+\mu}T_{fi}^k \\ +\displaystyle\sum_{k=1}^{4}c_i\dfrac{\lambda\psi_i i_k i_{ART}}{c_i+\lambda\psi_i i_k i_{ART}+\sigma_k+\varphi+\mu}A_{fi}^k \end{array}\right),$$

with $\lambda=\lambda_0$ before the intervention and in arm C, and $\lambda=\lambda_0 i_{bc}$ in arms A and B during the intervention (λ is the basic transmission rate, defined as the rate at which an untreated infected individual with CD4≥350 not in acute infection transmits to a partner, assuming they are both in the mid-risk group), and with the following population sizes, of adult males and females, stratified by risk group:

$$N_{mi}=\sum_{x=\{u,p,h,c\}}S_{mxi}+P_{mhi}+P_{mi}+\sum_{k=1}^{4}I_{mi}^k+\sum_{k=1}^{4}J_{mi}^k+\sum_{k=1}^{4}T_{mi}^k+\sum_{k=1}^{4}A_{mi}^k,$$

$$N_{mi}=S_{fi}+P_{fi}+\sum_{k=1}^{4}I_{fi}^k+\sum_{k=1}^{4}J_{fi}^k+\sum_{k=1}^{4}T_{fi}^k+\sum_{k=1}^{4}A_{fi}^k,$$

$$N_m=\sum_{i=1}^{3}N_{mi}\ ,\ N_f=\sum_{i=1}^{3}N_{fi}\ ,\ N=N_m+N_f\ .$$

Equations for the neighbouring communities

The equations governing the epidemic dynamics in the neighbouring communities are very similar, but with:

$$FOI_{ai}^{*}=c_i\left((1-\theta)\frac{\sum_{j=1}^{3}c_jK_{\bar{a}j}^{rand*}}{\sum_{j=1}^{3}c_jN_{\bar{a}j}^{*}}+\theta\frac{K_{\bar{a}i}^{assort*}}{N_{\bar{a}i}^{*}}\right)$$, with similar formulas for K_{ai}^{rand*} and $K_{ai}^{assort*}$ as for K_{ai}^{rand} and K_{ai}^{assort} and with population sizes calculated as for the clusters..

Model calibration

The model was calibrated to national HIV prevalence curves as reported by UNAIDS [27]. More precisely, the parameter values shown in Table S 9 were chosen to minimize the mean squared relative error between annual prevalence predicted by the model and UNAIDS prevalence estimates from 1990 to 2007. The fits for both countries are shown in Figure 3.

Table S 9. Fitted parameters. The model was calibrated to UNAIDS prevalence estimates for each country (see Figure 3).

Description	Name	Fitted Value (South Africa)	Fitted Value (Zambia)
Time of epidemic seeding	t_0	1979.50	1981.26
Basic transmission rate, defined as the rate at which an untreated infected individual with CD4≥350 not in acute infection transmits to a partner, assuming they are both in the mid-risk group	λ_0	0.23	0.39
Proportion in high risk group	f_{high}	0.23	0.18
Proportion in mid risk group	f_{mid}	0.30	0.22
Partner change rate in high risk group (year^{-1})	c_{high}	1.83	2.03
Assortativity of mixing	θ	0.87	0.90

Sensitivity analysis

- **Influence of parameter calibrated to prevalence curves**

We assessed whether the set of parameter values chosen to match UNAIDS prevalence estimates had a large impact on the predicted reduction in HIV incidence. To do so, we explored, using a Latin hypercube sampling scheme, a range of parameter values for the parameters described in Table S 9. We selected the 9 parameter sets (out of 9000) with best fits to the prevalence. For each of these, we then ran an optimization routine, starting from this parameter set, to obtain a neighbour parameter set with an even better fit to HIV prevalence. We compared the predicted reduction in incidence under these 9 final parameter sets to the original best-fit parameter combination. The 10 parameter sets are shown in Figure S 3 and S 4, and the corresponding fits and predicted HIV incidence and prevalence in Figure S 5. The relative reduction in HIV incidence in intervention arms compared to the control arms are shown in Figure S 6.

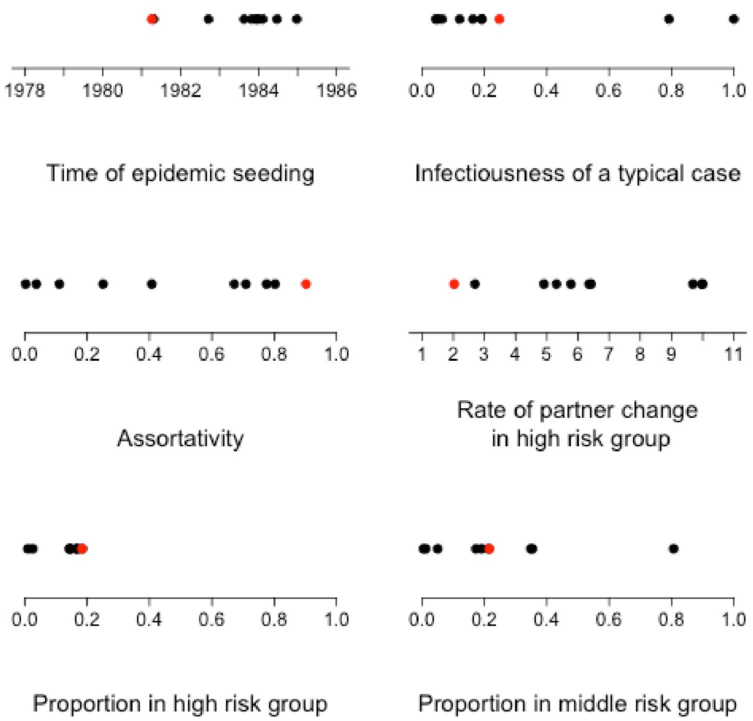

Figure S 3. Ten parameter sets calibrated to the UNAIDS prevalence estimates for Zambia. The red dots show the best fit parameter set.

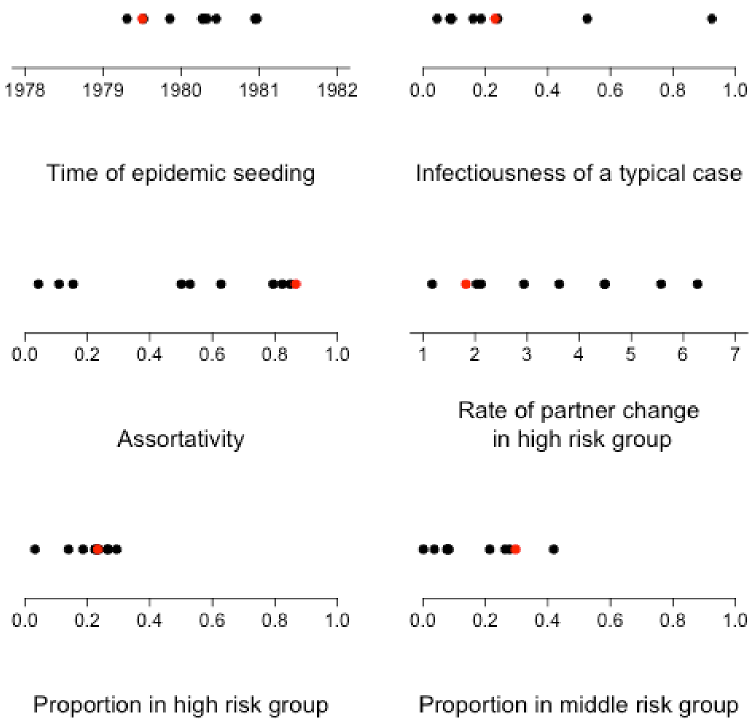

Figure S 4. Ten parameter sets calibrated to the UNAIDS prevalence estimates for South Africa. The red dots show the best fit parameter set.

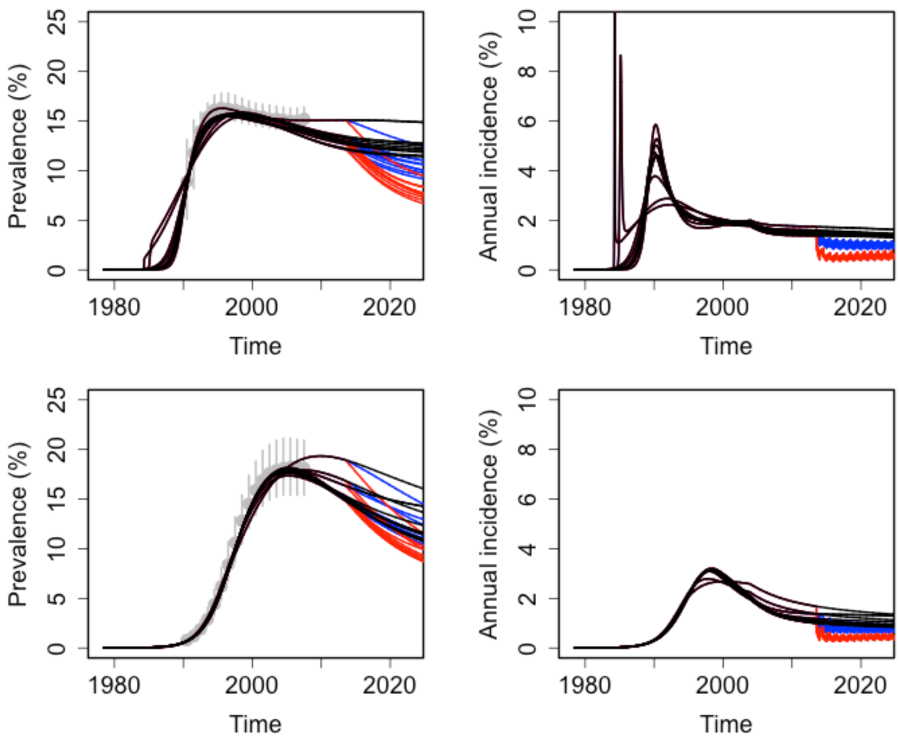

Figure S 5. Ten model fit and corresponding projections under central target scenario for Zambia (top row) and South Africa (bottom row). Left panels show HIV prevalence and right panels show HIV annual incidence. The red, blue and black lines correspond to arms A, B and C respectively. The grey dots and error bars are the UNAIDS prevalence estimates [27].

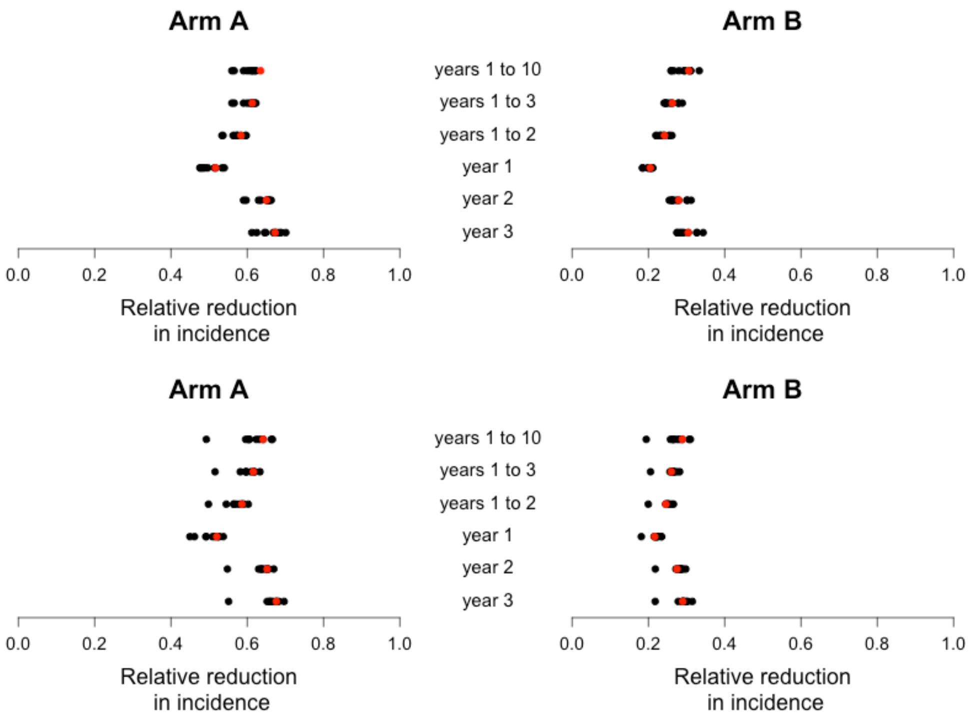

Figure S 6. Projected impact of the intervention on HIV incidence in Arms A and B compared with Arm C for central target scenario in Zambia (top row) and South Africa (bottom row), under 10 parameter sets calibrated to the UNAIDS prevalence estimates. The red dots show the best fit parameter set.

- **Influence of process parameters**

We explored the sensitivity of the main outcome (3-year cumulative HIV incidence) on process parameters (shown in Table 2) to anticipate which of those would need to be monitored most closely as they are most likely to influence the success of the trial. We defined a best-case and a worse-case value for each parameter (see Table S 10) during the intervention in arms A and B (assuming that before the intervention and in the control arm parameter values would be the "central target" ones, see Table S 10). For each country, we generated, using a Latin hypercube sampling scheme, a set of 1000 parameters drawn uniformly in those ranges and examined the resulting variability in the predicted 3-year cumulative HIV incidence in each arm.

We used a linear model to explore the relationship between the 3-year cumulative HIV incidence (on the natural scale) and the process parameters:

$$\Delta = \chi_0 + \chi_1 i_{ART} + \chi_2 i_{bc} + \chi_3 p_{test} + \chi_4 p_{ART} + \chi_5 \varphi_{UTT} + \chi_7 p_{circ} + \chi_8 i_{pART} + \chi_9 s_{pcirc} + \chi_{10} \tau_{circ} + \chi_{11} \upsilon_{wait} + \varepsilon$$

where Δ is the relative reduction in incidence (in either arm A or arm B compared to arm C), the χ_js are the coefficients of the regression, reported in Table 2, and ε is the error term.

The relative importance of each predictor was quantified by its contribution to the total variance. This was computed using the method described in [28], which performs a decomposition of the total variance that accounts for correlation between predictors.

Table S 10. Model parameters in four scenarios with various degrees of optimism

Parameter	Name	Most pessimistic target	Central target	Optimistic target	Most optimistic target	Ref
Proportion of sex acts with individuals from neighbouring areas	π	0.1	0.05	0.05	0.0	
Relative infectivity due to behavioural changes	i_{bc}	1.33	1.0	1.0	0.67	
Annual drop-out rate after intervention has started	φ_{UTT}	0.2	0.1	0.1	0.01	
Efficacy of ART in blocking transmission during intervention	i_{ART}	0.2	0.1	0.05	0.01	[21]
Rate of getting circumcised once decision to get circumcised has been made (year^{-1})	τ_{circ}	6 (average delay from test to circumcision: 2 months)	26 (average delay 2 weeks)	52 (average delay 1 week)	182.5 (average delay 2 days)	
Probability of going to get treatment given a positive HIV test delivered by CHiPs	p_{ART}	0.7	0.837	0.867	0.95	[13]
Probability of getting circumcised given negative HIV test delivered by CHiPs	p_{circ}	0.25	0.5	0.5	0.75	
Probability of accepting HIV test when offered by CHiPs	p_{test}	0.6	0.837	0.867	0.95	[13,14]
Relative infectivity of individuals waiting treatment	i_{pART}	1	1	1	0.8	Based on [29]
Relative susceptibility of individuals waiting circumcision	s_{pcirc}	1	1	1	0.8	Based on [29]
Mean time between positive test and actual start of treatment (year)	υ_{wait}	$\frac{2}{13}$	$\frac{1}{13}$	$\frac{3}{52}$	$\frac{8}{365}$	

- **Influence of the time period over which the intervention is carried out each year**

We simulated the intervention in arms A and B assuming that the intervention would be carried out over 6 months, 9 months or 12 months every year. We found that the relative reduction in cumulative 3-year incidence would be greater if the intervention was carried out over 6 months rather than 9 or 12 (see Figure S 7).

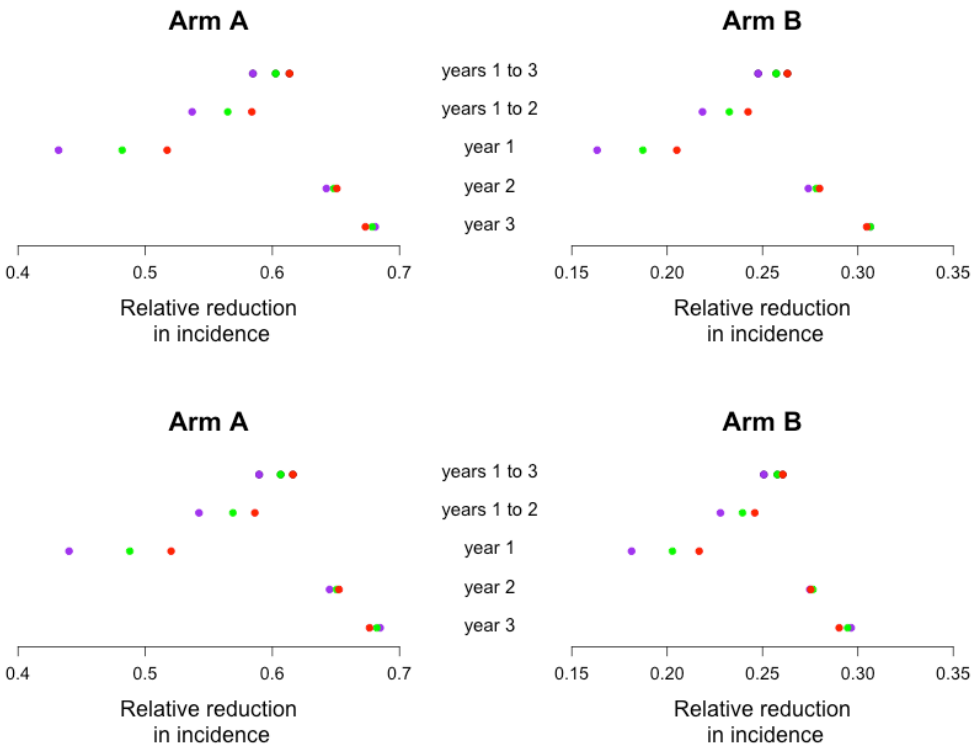

Figure S 7. Projected impact of the intervention on HIV incidence in Arms A and B compared with Arm C for central target scenario in Zambia (top row) and South Africa (bottom row), assuming the intervention is carried out over 6 months (red), 9 months (green) or 12 months (purple) every year.

References

1. Sismanidis C, Moulton LH, Ayles H, Fielding K, Schaap A, et al. (2008) Restricted randomization of ZAMSTAR: a 2 x 2 factorial cluster randomized trial. Clin Trials 5: 316-327.
2. Ayles HM, Sismanidis C, Beyers N, Hayes RJ, Godfrey-Faussett P (2008) ZAMSTAR, The Zambia South Africa TB and HIV Reduction Study: design of a 2 x 2 factorial community randomized trial. Trials 9: 63.
3. Lodi S, Phillips A, Touloumi G, Geskus R, Meyer L, et al. (2011) Time from human immunodeficiency virus seroconversion to reaching CD4+ cell count thresholds <200, <350, and <500 Cells/mm(3): assessment of need following changes in treatment guidelines. Clin Infect Dis 53: 817-825.
4. Hollingsworth TD, Anderson RM, Fraser C (2008) HIV-1 transmission, by stage of infection. J Infect Dis 198: 687-693.
5. Badri M, Bekker LG, Orrell C, Pitt J, Cilliers F, et al. (2004) Initiating highly active antiretroviral therapy in sub-Saharan Africa: an assessment of the revised World Health Organization scaling-up guidelines. AIDS 18: 1159-1168.
6. Thiebaut R, Pellegrin I, Chene G, Viallard JF, Fleury H, et al. (2005) Immunological markers after long-term treatment interruption in chronically HIV-1 infected patients with CD4 cell count above 400 x 10(6) cells/l. AIDS 19: 53-61.
7. May M, Boulle A, Phiri S, Messou E, Myer L, et al. (2010) Prognosis of patients with HIV-1 infection starting antiretroviral therapy in sub-Saharan Africa: a collaborative analysis of scale-up programmes. Lancet 376: 449-457.
8. Fernandez Guerrero ML, Rivas P, Molina M, Garcia R, De Gorgolas M (2005) Long-term follow-up of asymptomatic HIV-infected patients who discontinued antiretroviral therapy. Clin Infect Dis 41: 390-394.
9. Touloumi G, Pantazis N, Antoniou A, Stirnadel HA, Walker SA, et al. (2006) Highly active antiretroviral therapy interruption: predictors and virological and immunologic consequences. J Acquir Immune Defic Syndr 42: 554-561.
10. Gabillard D, Lewden C, Ndoye I, Moh R, Segeral O, et al. (2012) Mortality, AIDS-morbidity and loss to follow-up by current CD4 cell count among HIV-1 infected adults receiving antiretroviral therapy in Africa and Asia: data from the ANRS 12222 collaboration. J Acquir Immune Defic Syndr 62: 555-561.
11. Granich RM, Gilks CF, Dye C, De Cock KM, Williams BG (2009) Universal voluntary HIV testing with immediate antiretroviral therapy as a strategy for elimination of HIV transmission: a mathematical model. Lancet 373: 48-57.
12. Rosen S, Fox MP (2011) Retention in HIV care between testing and treatment in sub-Saharan Africa: a systematic review. PLoS Med 8: e1001056.
13. van Rooyen H, Barnabas RV, Baeten JM, Phakathi Z, Joseph P, et al. (2013) High HIV testing uptake and linkage to care in a novel program of home-based HIV counseling and testing with facilitated referral in KwaZulu-Natal, South Africa. J Acquir Immune Defic Syndr 64: e1-8.

14. Sabapathy K, Van den Bergh R, Fidler S, Hayes R, Ford N (2012) Uptake of Home-Based Voluntary HIV Testing in Sub-Saharan Africa: A Systematic Review and Meta-Analysis. PLoS Med 9: e1001351.
15. Kigozi G, Gray RH, Wawer MJ, Serwadda D, Makumbi F, et al. (2008) The safety of adult male circumcision in HIV-infected and uninfected men in Rakai, Uganda. PLoS Med 5: e116.
16. Matthews GV, Sabin CA, Mandalia S, Lampe F, Phillips AN, et al. (2002) Virological suppression at 6 months is related to choice of initial regimen in antiretroviral-naive patients: a cohort study. AIDS 16: 53-61.
17. Garnett GP, Anderson RM (1993) Factors controlling the spread of HIV in heterosexual communities in developing countries: patterns of mixing between different age and sexual activity classes. Philos Trans R Soc Lond B Biol Sci 342: 137-159.
18. Powers KA, Ghani AC, Miller WC, Hoffman IF, Pettifor AE, et al. (2011) The role of acute and early HIV infection in the spread of HIV and implications for transmission prevention strategies in Lilongwe, Malawi: a modelling study. Lancet 378: 256-268.
19. Wawer MJ, Makumbi F, Kigozi G, Serwadda D, Watya S, et al. (2009) Circumcision in HIV-infected men and its effect on HIV transmission to female partners in Rakai, Uganda: a randomised controlled trial. Lancet 374: 229-237.
20. Mehta SD, Gray RH, Auvert B, Moses S, Kigozi G, et al. (2009) Does sex in the early period after circumcision increase HIV-seroconversion risk? Pooled analysis of adult male circumcision clinical trials. AIDS 23: 1557-1564.
21. Cohen MS, Chen YQ, McCauley M, Gamble T, Hosseinipour MC, et al. (2011) Prevention of HIV-1 infection with early antiretroviral therapy. N Engl J Med 365: 493-505.
22. Auvert B, Taljaard D, Lagarde E, Sobngwi-Tambekou J, Sitta R, et al. (2005) Randomized, controlled intervention trial of male circumcision for reduction of HIV infection risk: the ANRS 1265 Trial. PLoS Med 2: e298.
23. Gray RH, Kigozi G, Serwadda D, Makumbi F, Watya S, et al. (2007) Male circumcision for HIV prevention in men in Rakai, Uganda: a randomised trial. Lancet 369: 657-666.
24. Bailey RC, Moses S, Parker CB, Agot K, Maclean I, et al. (2007) Male circumcision for HIV prevention in young men in Kisumu, Kenya: a randomised controlled trial. Lancet 369: 643-656.
25. Mills E, Cooper C, Anema A, Guyatt G (2008) Male circumcision for the prevention of heterosexually acquired HIV infection: a meta-analysis of randomized trials involving 11,050 men. HIV Med 9: 332-335.
26. Diekmann O, Heesterbeek JAP (2000) Mathematical Epidemiology of Infectious Diseases. New York: Wiley.
27. UNAIDS/WHO (2008) Epidemiological Country Profiles - HIV & AIDS 2008.
28. Gromping U (2006) Relative importance for linear regression in R: The package relaimpo. Journal of Statistical Software 17.
29. Shafer LA, Nsubuga RN, White R, Mayanja BN, Chapman R, et al. (2011) Antiretroviral therapy and sexual behavior in Uganda: a cohort study. AIDS 25: 671-678.
